# Probing entanglement scaling across a quantum phase transition on a quantum computer

**DOI:** 10.1038/s41467-026-75251-x

**Published:** 2026-07-27

**Authors:** Qiang Miao, Tianyi Wang, Kenneth R. Brown, Thomas Barthel, Marko Cetina

**Affiliations:** 1 Duke Quantum Center, Duke University, Durham, North Carolina USA; 2 Department of Physics, Duke University, Durham, North Carolina USA; 3 Department of Electrical and Computer Engineering, Duke University, Durham, North Carolina USA; 4 Department of Chemistry, Duke University, Durham, North Carolina USA; 5 National Quantum Laboratory, University of Maryland, College Park, MD USA; 6 Department of Physics, University of Maryland, College Park, MD USA

**Keywords:** Quantum simulation, Quantum information

## Abstract

The investigation of strongly-correlated quantum matter is difficult due to the curse of dimensionality and intricate entanglement structures. These challenges are particularly pronounced in the vicinity of continuous quantum phase transitions, where quantum fluctuations manifest across all length scales. While quantum simulators give controlled access to a number of strongly correlated systems, the study of critical phenomena has been hampered by finite-size effects arising from diverging correlation lengths. Moreover, the experimental investigation of entanglement in many-body systems has been hindered by limitations in measurement protocols. To address these challenges, we employ the multiscale entanglement renormalization ansatz (MERA) and implement a holographic scheme for subsystem tomography on a fully-connected trapped-ion quantum computer. Our method accurately represents infinite systems and long-range correlations with few qubits, facilitating the efficient extraction of observables and entanglement properties, even at criticality. We observe a quantum phase transition with spontaneous symmetry breaking and reveal the evolution of entanglement properties across the critical point. For the first time, we demonstrate log-law scaling of subsystem entanglement entropies at criticality on a digital quantum computer. This achievement highlights the potential of MERA for the investigation of strongly-correlated many-body systems on quantum computers.

## Abstract (web summary)

The authors implement a tensor network framework to encode states of large quantum systems with few trapped-ion qubits. This enables clear observation of a quantum phase transition and a transition in the scaling of subsystem entanglement entropies.

## Introduction

In physics and materials science, universality appears near continuous phase transitions, where long-range properties become independent of microscopic details, and the singular behavior of physical quantities such as susceptibilities and correlation lengths can be characterized by universal critical exponents. While classical phase transitions are driven by thermal fluctuations, quantum phase transitions occur at zero temperature when a system parameter is varied, causing the ground state to become highly entangled due to quantum fluctuations^1,2^. This ground-state non-locality presents challenges for theoretical and experimental analysis.

Rapid advancements on controlled quantum platforms have expanded the range of experimentally accessible quantum many-body systems on quantum simulators and computers^3–10^. Previous studies^11–18^ have made significant progress in the experimental characterization of entanglement. However, probing entanglement properties across quantum phase transitions remains difficult. Analog quantum simulators have limited control, restricting available models and measurements^19^. Quantum simulations using quantum computers face challenges, including: (i) Limited number of qubits: Quantum phase transitions formally occur only in infinite systems, and finite-size effects are pronounced in the vicinity of critical points. Preparing critical ground states directly requires more qubits than current quantum devices offer. (ii) Exponential cost for probing subsystem entanglement: Resolving detailed entanglement structures requires state tomography for large subsystems, which generally results in exponential costs. (iii) Difficulties in state preparation: Without deeper insight into the physics, the study of complex many-body ground states necessitates highly expressive quantum circuits with many gates, leading to low trainability^20,21^ and significant error accumulation.

We overcome these challenges for condensed-matter ground states using highly structured quantum circuits on a digital quantum computer. The circuits prepare states corresponding to hierarchical tensor networks—the multiscale entanglement renormalization ansatz (MERA)^22–24^. This ansatz can represent infinite systems and accurately encodes long-range correlations, even at critical points. The causal structure of MERA, inspired by the real-space renormalization group, drastically reduces circuit depths and the number of measured qubits, both scaling logarithmically with system size^25–28^. This allows efficient extraction of local observables, subsystem entanglement, and entanglement spectra. Additionally, MERA is robust to noise^29^ and not affected by barren plateaus^30,31^.

In this work, we study quantum many-body ground states on a digital quantum computer based on a chain of ytterbium ions. We demonstrate a quantum phase transition characterized by spontaneous symmetry breaking in the thermodynamic limit, using 12 or fewer qubits. We show the universal scaling of relevant physical quantities near the transition and observe significant changes in the entanglement structure across the critical point. At the critical point, we find that the subsystem entanglement entropies follow a universal log-area law, and the gap of the entanglement Hamiltonian closes as the subsystem size increases. In non-critical regimes, subsystem entanglement entropy follows an area law^32–34^. The measured entanglement Hamiltonian remains gapped with increasing subsystem size and accurately reproduces expected symmetry properties.

## Results

Preparing ground states of many-body systems with long-range quantum fluctuations is challenging. This can be mitigated by accounting for the system’s entanglement structure. To approximate the ground state $\left\vert \Psi \right\rangle$ in the thermodynamic limit, we implement MERA on a digital ion-trap quantum computer.

### MERA circuits

**Fig. 1:**
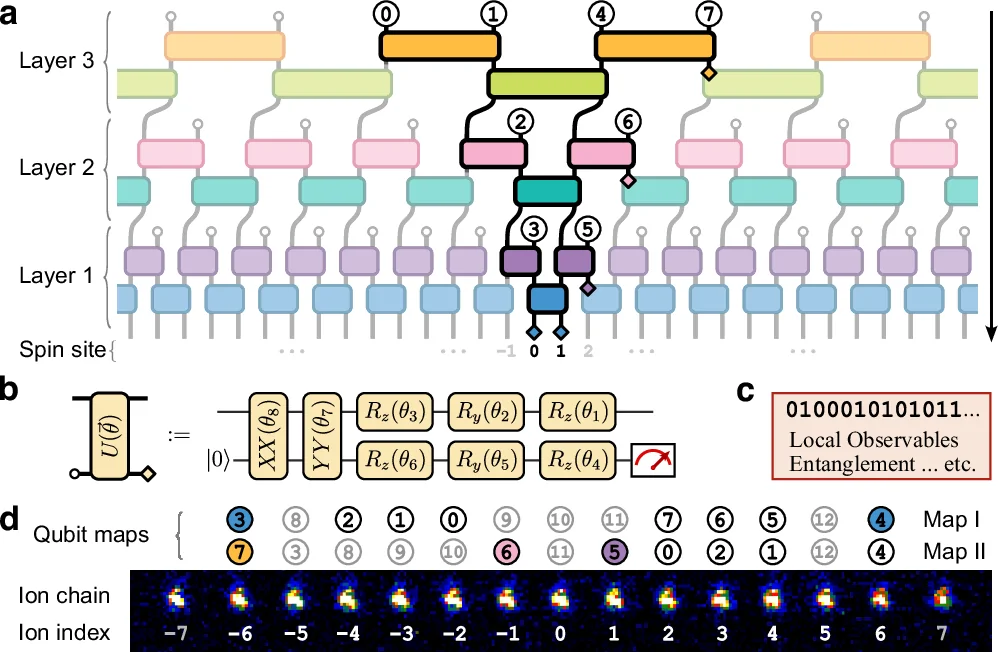
MERA on a trapped-ion chain. **a** An infinite, binary 1D MERA with three homogeneous layers. The ansatz is prepared from top to bottom as indicated by the arrow, with each layer comprising entangling gates (bare rectangles) and isometries (rectangles with attached circles) that double the number of sites. The output is the many-body state on the physical spin sites. Repeating the MERA circuit horizontally in the spatial dimension generates a state in the thermodynamic limit. Local observables and bipartite entanglement can be studied by executing circuits within the corresponding causal cone (unshaded area) and measuring the target qubits. Circles represent qubits initialized in $\left\vert 0\right\rangle$, and diamonds indicate measurements. **b** Each MERA tensor is implemented as a sequence of two- and one-qubit gates. **c** Repeated preparation and measurement yield probabilistic binary outcomes (0 s and 1 s) from which the desired physical properties can be inferred. **d** Fluorescence image of a 15-ion (^171^Yb^+^) chain with two hardware-tailored examples of ion-to-qubit mappings: Map I for local measurements on sites 0 and 1, and Map II optimized for entanglement studies with respect to a bipartition into the semi-infinite subsystems *A* = ( − *∞*, 1] and *B* = [2, *∞*). Each qubit is represented by a circle and identified by the number inside. Active qubits in the MERA causal-cone circuit are outlined in black, while unused qubits are shown in gray (shaded). For active qubits, a colored background indicates that the qubit is to be measured, with the color corresponding to the last tensor acting on it.

As shown in Fig. 1a, the layered structure of MERA tensor networks introduces an additional dimension associated with a hierarchy of length and energy scales. The MERA comprises *T* layers, each composed of unitary (dis)entanglers and isometries, which can be efficiently implemented on our hardware. Viewed from bottom to top, starting from the physical layer, MERA acts as a renormalization group (RG) flow, where the ascending layer index represents the RG scale. In each renormalization step, short-distance entanglement is removed by the disentanglers (bare rectangles), and the isometries (rectangles with attached circles) then coarse-grain the system. This process maps physical sites to renormalized effective degrees of freedom, continuing until the final layer $T \sim \log \xi$ is reached, where *ξ* is the largest correlation length in the system.

Viewed in reverse (the direction of decreasing layer index), the MERA represents a quantum circuit, which prepares the quantum state by first generating long-range correlations associated with low-energy modes, and then progressively incorporating shorter-range correlations that correspond to higher energy scales. For each layer, ancillary qubits initialized in the $\left\vert 0\right\rangle$ state (depicted as circles) are injected and entangled with existing qubits through unitary operations. Each unitary tensor consists of two-qubit entangling gates and single-qubit rotations, as illustrated in Fig. 1b.

### Observables and holographic subsystem tomography

Local observables can be studied by implementing only the associated causal-cone circuits. For measurements on sites 0 and 1 of a binary one-dimensional (1D) MERA, the corresponding reduced circuit is indicated by the non-shaded MERA tensors in Fig. 1a. Due to their isometric properties, the shaded tensors outside the causal cone do not influence the local measurements.

Entanglement entropies and spectra for a spatial bipartition of the system into parts *A* and *B* can be obtained from the reduced density matrix ${\hat{\varrho }}_{B}={{{{\rm{Tr}}}}}_{A}\vert \Psi \rangle \langle \Psi \vert$. For a MERA, only the gates within the causal cone of the boundary between *A* and *B* need to be implemented, as gates outside the causal cone correspond to unitary basis transformations ${\hat{U}}_{A}$ and ${\hat{U}}_{B}$ in the two subsystems and do not affect bipartite entanglement properties. Moreover, tomography on the renormalized sites (qubits) at the edges of *A*’s causal cone provides access to ${\hat{\varrho }}_{B}$ in the basis defined by ${\hat{U}}_{B}$. Thus, due to the causal structure of MERA, the number of gates and number of measured qubits are proportional to the number of layers $T \sim \log \xi$, rather than the total system size and subsystem size, respectively. Details of our scheme, termed *holographic subsystem tomography*, are described in “Methods”.

In the experiment, we implement an infinite, homogeneous, binary 1D MERA with bond dimension *χ* = 2 and up to *T* = 5 layers, which captures a maximum correlation range of 3 × 2^5^ = 96 sites. To this purpose, all tensors of the same color in Fig. 1a are chosen to be identical, and the shown tensor network structure is continued ad infinitum horizontally. For the self-similar critical systems, the correlation length *ξ* diverges, which we address by employing a *scale-invariant MERA*^35,36^ (see “Methods”).

### Trapped-ion system

We implement MERA circuits on a digital ion-trap quantum computer. Our experimental system consists of a linear chain of fifteen ^171^Yb^+^ ions, spaced by approximately 3.7 μm, confined in a micro-fabricated Paul trap (Sandia HOA-2.1.1^37^) held in a room-temperature vacuum chamber. The qubits are encoded in the hyperfine “clock” states of the ground ^2^S_1/2_ electronic manifold as $\left\vert 0\right\rangle \equiv \left\vert F=0;{m}_{F}=0\right\rangle$ and $\left\vert 1\right\rangle \equiv \left\vert F=1;{m}_{F}=0\right\rangle$, where *F* is the total atomic angular momentum and *m*_*F*_ its projection onto the direction of the magnetic field, with a magnitude of 3.2 G. Qubits are initialized in the $\left\vert 0\right\rangle$ state using optical pumping and measured by state-dependent fluorescence on the 369 nm D1 line.

The qubits are manipulated via a Raman process using a wide (300 μm × 30 μm) linearly-polarized global-addressing beam and equispaced, linearly-polarized 1 μm-diameter individual-addressing beams derived from the same 355-nm pulsed laser (Coherent Paladin Compact 355-4000). Radio-frequency (RF) gate waveforms are produced by an RF system on a chip (ZCU111 from Xilinx Corp.) controlled by the Sandia Octet firmware^38^ and separately modulated onto each laser beam using acousto-optic modulators (L3 Harris Corp.). Available single-qubit operations are virtual *z*-axis rotations *R*_*z*_(*θ*), implemented via phase offsets in the radio-frequency controls, and physical *xy*-plane rotations $R(\theta,\phi )={{{{\rm{e}}}}}^{-{{{\rm{i}}}}\theta (\hat{X}\cos \phi+\hat{Y}\sin \phi )/2}$. Entangling gates $XX(\theta )={{{{\rm{e}}}}}^{-{{{\rm{i}}}}\theta {\hat{X}}_{i}\otimes {\hat{X}}_{j}/2}$ between arbitrary qubits *i* and *j* are realized through a Mølmer-Sørensen interaction mediated by the shared radial motional modes of the ion chain with mode frequencies near 3 MHz. This interaction is implemented by amplitude-shaping a state-dependent force on the addressed ions using the 355-nm Raman process.

The all-to-all qubit connectivity of our platform simplifies the implementation of the non-local MERA circuits. Using a detailed error model discussed in “Methods”, we determine the ion-to-qubit mapping for each circuit based on the estimated error in the target observables. We suppress crosstalk by assigning the measured qubits to the non-adjacent ions and placing higher-fidelity gates closer to the final measurement operations. Based on these principles, in the example of Fig. 1d, we chose Map I for measuring local observables and Map II for measuring entanglement entropy.

### Model

To demonstrate the potential of the MERA approach, we apply it to the 1D transverse-field Ising model (TFIM) described by the Hamiltonian

1 $$\hat{H}=-{\sum}_{i}^{L}{\hat{X}}_{i}{\hat{X}}_{i+1}-g{\sum}_{i}^{L}{\hat{Z}}_{i}.$$

Here, ${\hat{X}}_{i}$ and ${\hat{Z}}_{i}$ are Pauli operators acting on the spin at lattice site *i*, and *g* is the transverse magnetic field. The TFIM features a well-characterized continuous quantum phase transition with spontaneous ${{\mathbb{Z}}}_{2}$ symmetry-breaking, making it a good testbed for our approach. The gate compilation of the corresponding MERA tensors and a concrete circuit example are discussed in Supplementary Note 1.

Since the 1D binary MERA with a small bond dimension is classically simulable, we determine the optimal gate parameters of the variational MERA circuit using classical computers. However, a full hybrid optimization with quantum hardware is also practically feasible (see Supplementary Note 2) and will become necessary when scaling to more complex, higher-dimensional physical models.

### Quantum phase transition

We benchmark the infinite MERA across the quantum phase transition of the TFIM (1) by first measuring local observables $\langle {\hat{O}}_{i}\rangle$. We prepare the causal-cone state of the target spins as indicated in Fig. 1. The number *T* of employed MERA layers ranges from 2–5, depending on the correlation length; *T* = 2 layers are used far from criticality and up to 5 layers near the critical point *g* = 1. Correspondingly, the number of required qubits ranges from 6–12. With these *T* and bond dimension *χ* = 2, the numerically optimized MERA already reaches an energy density precision of  ≲ 10^−3^. Thus, unless otherwise noted, the leading errors arise from experimental noise, as we show below.

**Fig. 2:**
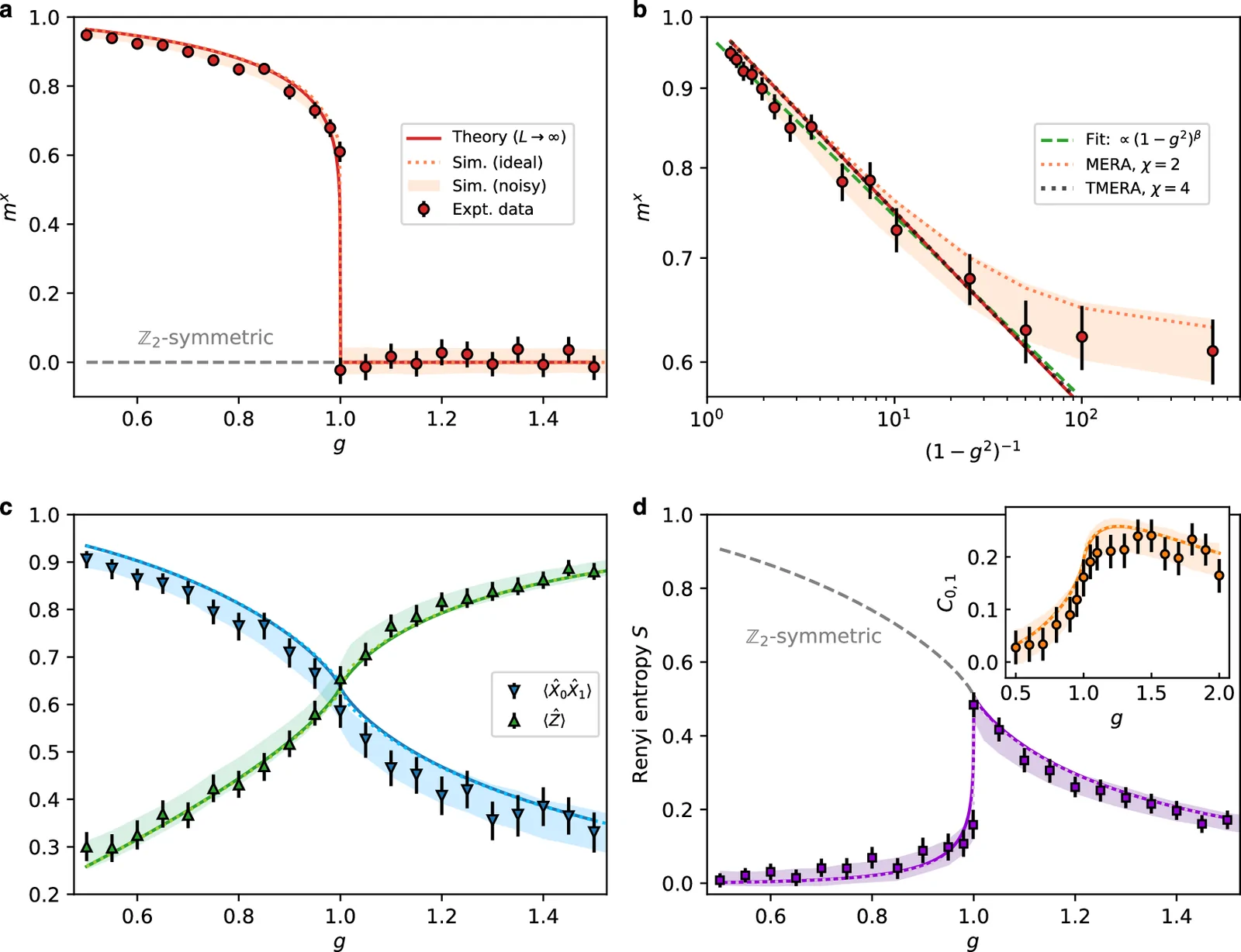
Local observables and few-site entanglement in the 1D transverse-field Ising model as a function of the field *g.* **a** Mean magnetization *m*^*x*^ over spin sites 0 and 1. The dashed gray line represents the reference value for a ${{\mathbb{Z}}}_{2}$-symmetric state. **b** Logarithmic plot of *m*^*x*^ in the ${{\mathbb{Z}}}_{2}$ symmetry-broken ferromagnetic phase (*g* < 1). The green dashed line represents a fit to the experimental data, excluding the last two points, yielding an extracted critical exponent of *β* = 0.117(4). For comparison, the exact theoretical value for the Ising class is 1/8 = 0.125 (solid red line). The orange and gray dotted lines show the numerical predictions for MERA with bond dimension *χ* = 2 and Trotterized MERA^25,26^ with *χ* = 4, respectively. **c** Local observables corresponding to the two competing terms in the Hamiltonian (1). **d** The Rényi-2 entanglement entropy of a single spin. The dashed gray line corresponds to the ${{\mathbb{Z}}}_{2}$-symmetric ground state. The inset shows the nearest-neighbor concurrence *C*_0,1_ (pairwise entanglement) for two neighboring sites. For all panels, solid lines correspond to theoretical predictions in the thermodynamic limit, dotted lines represent ideal infinite MERA simulations, and the shaded areas indicate the central 95% quantile obtained from noisy simulations based on an error model that reflects experimental device characteristics. Error bars capture statistical errors, indicating 95% confidence intervals obtained from a bootstrap resampling of the experimental data, based on 2000 measurements per quantum circuit.

Figure 2a shows the measured magnetization *m*^*x*^ in the *x*-direction as a function of the *z*-field *g*, nicely resolving the phase transition. For *g* > 1, the observed magnetization is consistent with zero within the statistical uncertainty, as expected for the paramagnetic phase. For *g* < 1, non-zero magnetization is observed along the *x*-direction, indicating spontaneous breaking of the $\hat{X}\to -\hat{X}$ symmetry, characteristic of the ferromagnetic phase. In the thermodynamic limit (*L* → *∞*), this phase features two degenerate ground states with opposite spin alignments. Rather than converging to a superposition, the variational optimization of the MERA spontaneously selects one of these symmetry-broken states depending on the random initialization.

Due to experimental noise, the observed magnetization at small *g* is slightly lower than the theoretical prediction ${(1-{g}^{2})}^{1/8}$^39–41^. We conducted separate experiments to characterize noise and develop a corresponding error model, as described in “Methods”. In simulations, we model gate-induced dephasing and addressing noise due to ion motion by randomly perturbing gates in each shot. We model idle dephasing, state preparation and measurement (SPAM) errors, and *X*-flip errors during entangling gates by introducing random *Z*- and *X*-flips. The noisy simulations (shaded area in Fig. 2a) demonstrate excellent agreement with experimental data.

To determine the critical exponent, we plot the measured order parameter *m*^*x*^ as a function of ${(1-{g}^{2})}^{-1}$ (Fig. 2b). For ${(1-{g}^{2})}^{-1} < 80$, i.e., *g* < 0.994, the experimental data follows the expected power law (solid red line). A fit of the measured *m*^*x*^ in this regime to the form $A\,{(1-{g}^{2})}^{\beta }$, taking into account the statistical errors, yields the critical exponent of *β* = 0.117(4). This value is close to the theoretical prediction of 1/8 = 0.125 for the Ising universality class. As the system enters the immediate vicinity of the critical point (${(1-{g}^{2})}^{-1} > 20$), numerical simulations (orange dotted line) show that *m*^*x*^ saturates at a non-zero value. This deviation arises from the small MERA bond dimension *χ* = 2, and can be mitigated by increasing the bond dimension *χ*. As shown by the thick gray dotted line, a Trotterized MERA circuit^25–28^ with *χ* = 4 is predicted to achieve sufficient accuracy to capture the critical behavior even in this regime, at the cost of more qubits and gates. Figure 2c shows the local observables that constitute the Hamiltonian (1). As the transverse field *g* increases, the dominant contribution to the groundstate energy shifts from the correlator $\langle {\hat{X}}_{i}{\hat{X}}_{i+1}\rangle$ to the field term $\langle {\hat{Z}}_{i}\rangle$, showing a symmetry due to the Kramers-Wannier duality^42^ with respect to the critical point *g* = 1. The measured values of these observables cross at a slightly smaller value of *g*, aligning with our noise model. Here, the noise is dominated by fluctuations in *XX*-gate rotations due to the ions’ axial motion (see “Methods”).

### Entanglement structure

In the following, we use MERA to investigate ground-state entanglement across the quantum phase transition in the TFIM (1). We find that MERA on the digital quantum computer can resolve details of the entanglement structure, including the hallmark transition from area-law to log-area-law scaling of block entanglement entropies when approaching the critical point.

(i) Local entanglement measures: For a pure state $\left\vert \Psi \right\rangle$, the entanglement between subsystem *B* and its complement *A* can be quantified by the Rényi entanglement entropy

2 $${S}_{B}^{(\alpha )}=\frac{1}{1-\alpha }{\log }_{2}{{{\rm{Tr}}}}({\hat{\varrho }}_{B}^{\alpha }),\,\,{{{\rm{where}}}}\,\,{\hat{\varrho }}_{B}={{{\rm{Tr}}}}_{A}\vert \Psi \rangle \langle \Psi \vert$$

is the reduced density matrix of subsystem *B*. Our approach provides efficient access to the entanglement spectra and, hence, ${S}_{B}^{(\alpha )}$ for any *α*. Here, we focus on the Rényi-2 entropy (*α* = 2) as, for the TFIM (1), all Rényi entropies with *α* > 0 exhibit the same scaling.

The reduced density matrix for a single site is fully characterized by its Bloch vector. The time-reversal symmetry of the TFIM ensures that the local expectation value $\langle \widehat{Y}\rangle$ vanishes for all eigenstates. Thus, the Bloch vector is determined by $\langle \hat{X}\rangle$ and $\langle \hat{Z}\rangle$. Figure 2d shows the measured single-site Rényi-2 entanglement entropy. Our data faithfully reproduces the predicted sharp peak at criticality and tends to zero for small *g*. In the ferromagnetic phase, the two symmetry-broken ground states are almost product states. Meanwhile, their symmetric superposition is entangled, and the corresponding single-site entanglement is smooth at the critical point.

The inset of Fig. 2d presents the nearest-neighbor concurrence (pairwise entanglement) *C*_0,1_^41,43^ obtained from the measured nearest-neighbor correlators $\langle {\hat{X}}_{0}{\hat{X}}_{1}\rangle$, $\langle {\hat{Z}}_{0}{\hat{Z}}_{1}\rangle$, and $\langle {\hat{Y}}_{0}{\hat{Y}}_{1}\rangle$^44,45^. The concurrence slightly deviates from theoretical predictions but aligns well with noisy simulations. Notably, the concurrence does not peak at criticality but increases smoothly before reaching a broad maximum in the paramagnetic phase. This suggests that the peak of single-site entanglement at criticality arises not only from short-distance entanglement but also from the accumulation of entanglement over long distances. In contrast, in the paramagnetic phase, the nearest-neighbor entanglement is the dominant contribution to the single-site entanglement.

(ii) Area-law versus log-area-law: For the ground states of typical quantum many-body systems in *D* spatial dimensions, the subsystem entanglement entropy *S*(*ℓ*) is proportional to the surface area of the subsystem ∝ *ℓ*^*D*−1^, rather than its volume ∝ *ℓ*^*D*^, where *ℓ* denotes the linear subsystem size^32–34^. The entanglement entropy is dominated by short-range correlations around the subsystem boundary, resulting in area-law scaling. This can change when the system becomes critical, and the correlation length diverges. For critical systems in 1D and fermionic systems with a Fermi surface of codimension *D* − 1, the subsystem entanglement entropy is predicted to follow a log-area law $\propto {\ell }^{D-1}\log \ell$^46–51^.

Resolving these fundamentally different scaling laws has remained an experimental challenge. In small systems, it is difficult to differentiate between log-area-law and area-law scalings. As the linear subsystem size *ℓ* grows, preparing ground states with sufficient precision becomes increasingly difficult, and, in direct approaches, the number of measurements needed for subsystem tomography grows exponentially in *ℓ*.

For MERA, however, each step of the renormalization process changes length scales by a constant factor. This maps a logarithmic scaling in the subsystem size *ℓ* to a linear scaling in the number of renormalization steps *T* = 1, 2, 3, …. Thus, distinguishing between area and log-area laws in 1D translates to determining whether the entanglement entropy saturates or grows linearly with increasing *T*.

We experimentally realize a spatially infinite MERA with a finite number of layers *T*. For a bipartition into subsystems *A* = ( − *∞*, 1] and *B* = [2, + *∞*), we implement the holographic subsystem tomography as described above and detailed in “Methods”. We prepare the MERA circuit within the causal cone of the subsystem boundary, measure the *T* renormalized sites at the right edge of the cone (Fig. 1), and use the classical shadow method^52^ to extract the Rényi-2 entanglement entropy (2). The finite number of MERA layers limits the maximal correlation length to 3 × 2^*T*^. Thus, we experimentally probe effective subsystem sizes *ℓ* ∝ 2^*T*^. See “Methods” for a more detailed argument on this relation of *T* and effective subsystem sizes.

**Fig. 3:**
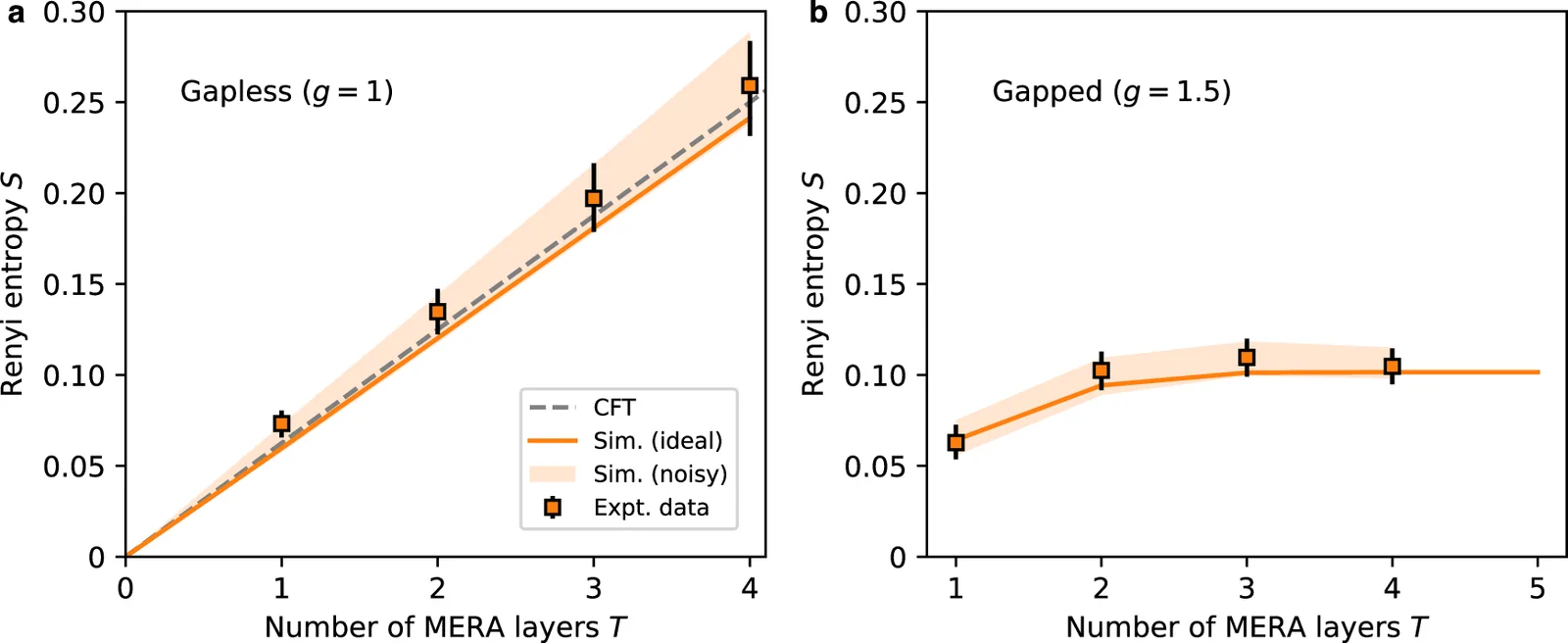
Log-area-law and area-law scaling of entanglement entropy for critical and gapped systems. **a** Bipartite half-chain Rényi-2 entanglement entropy for the infinite Ising chain (1) at the critical point *g* = 1, depending on the number *T* of MERA layers. The effective subsystem size is *ℓ* = 3 × 2^*T*^. The gray dashed line shows the log-area law prediction of conformal field theory (CFT) with central charge *c* = 1/2, and the solid line corresponds to ideal MERA simulations. Both the noisy simulation (shaded area) and experimental data (dots with error bars) are shown with 95% confidence intervals based on 1500 shots per element of the tomographic measurement basis. **b** Rényi-2 entanglement entropy for MERA states at the non-critical point *g* = 1.5. Here, the half-chain entanglement saturates rapidly as the number *T* of MERA layers increases, indicating the area law. The shaded area and error bars show 95% confidence intervals based on 8000, 5000, 3000, and 2000 shots per measurement basis element for cases with one-, two-, three-, and four-layer MERA, respectively.

For the non-critical point *g* = 1.5 of the TFIM (1), the measured half-chain Rényi-2 entanglement entropy *S* is shown in Fig. 3b. We observe a rapid saturation of the entanglement entropy for *T*≥3, in agreement with the area-law. This is expected as, due to the energy gap, the physical correlation length *ξ* is finite. Here, additional MERA layers (*T* > 3) have a negligible effect on the ground-state approximation and entanglement for this gapped system. Our error model indicates that, in this regime, SPAM errors and gate rotation errors due to the ions’ axial motion are the dominant noise sources.

At criticality (*g* = 1), the system has gapless excitations, and the correlation length diverges (*ξ* → *∞*). Here, we model the ground state using a scale-invariant MERA with an infinite number of homogeneous identical layers in both the spatial and preparation directions^35,36^. Directly preparing a scale-invariant MERA is not experimentally feasible. Instead, we truncate at layer *T* and add an additional tensor to the top layer to minimize the finite-*T* effects as detailed in “Methods”.

The measured half-chain Rényi-2 entanglement entropy *S* at criticality is shown as a function of *T* in Fig. 3a. The observed entropy increases linearly by approximately 1/16 per MERA layer. This is consistent with the prediction $\frac{1}{16}{\log }_{2}(\ell /a)$ of conformal field theory, where *a* is an ultraviolet cutoff that regularizes the continuum field theory^53^. The slight increase in the measured *S* over the field-theoretical prediction is reproduced by our error model. Here, errors primarily arise during SPAM and from fluctuations in gate angles due to the ions’ axial motion; *X*-flips and idle dephasing are secondary (see “Methods”).

(iii) Entanglement spectrum: Using the holographic tomography data and a maximum-likelihood approach^54–57^, we reconstruct proper (positive semidefinite) density matrices ${\hat{\varrho }}_{B}$ for subsystem *B* in the ${\hat{U}}_{B}$ basis. They can be written in the form ${\hat{\varrho }}_{B}={2}^{-{\sum }_{i}{\zeta }_{i}\left\vert {\zeta }_{i}\right\rangle \left\langle {\zeta }_{i}\right\vert }$, where $\{{\zeta }_{i}:=-{\log }_{2}{\lambda }_{i}\}$ is the entanglement spectrum^58^ expressed in terms of the eigenvalues {*λ*_*i*_} of ${\hat{\varrho }}_{B}$.

**Fig. 4:**
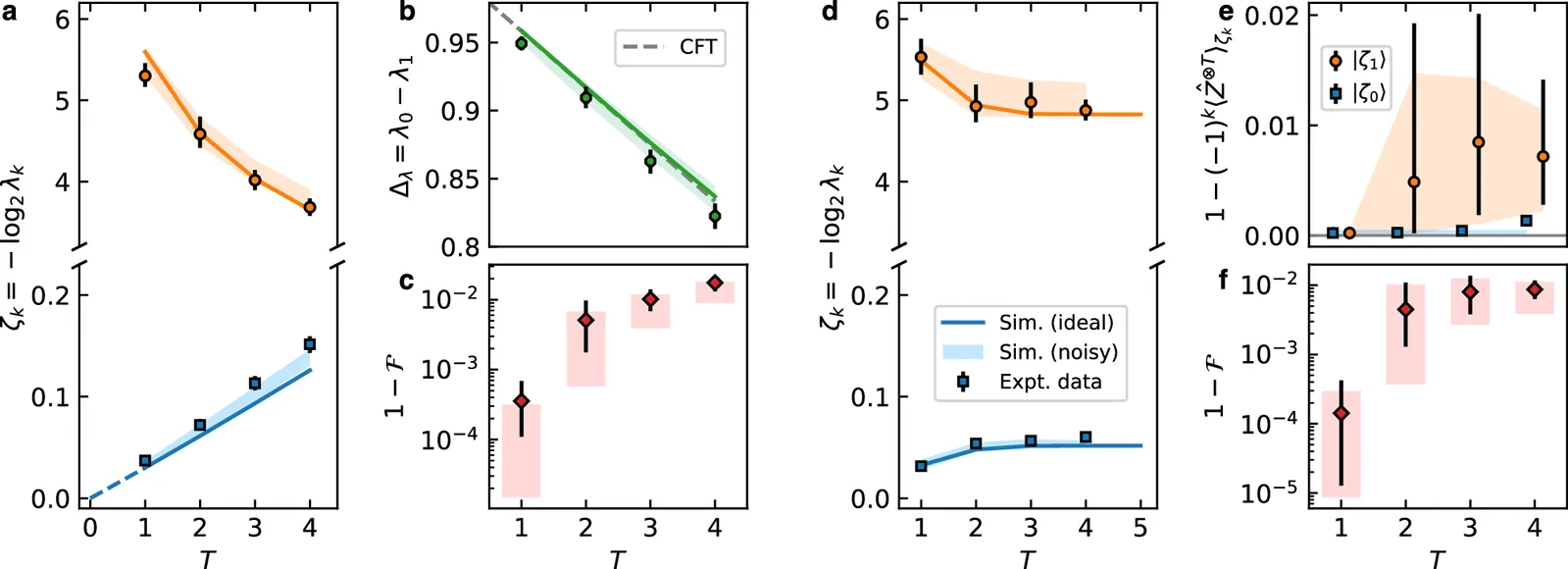
Analysis of half-chain density matrices. **a**, **d** *T*-dependence of the lowest two eigenvalues {*ζ*_0_, *ζ*_1_} of the MERA entanglement Hamiltonian at the critical point *g* = 1 and the non-critical point *g* = 1.5, respectively. **b** The Schmidt gap *Δ*_*λ*_ = *λ*_0_ − *λ*_1_ gradually closes as the (sub)system size increases at criticality. The gray dashed line represents the theoretical approximation from ref. ^59^. **c**,** f** Infidelity of the subsystem density matrices relative to ideal simulations at *g* = 1 and *g* = 1.5, respectively. **e** Expectation values of Pauli *Z*-strings for the first two eigenstates $\{\left\vert {\zeta }_{0}\right\rangle,\left\vert {\zeta }_{1}\right\rangle \}$ of the entanglement Hamiltonian at *g* = 1.5. In all plots, solid lines represent ideal infinite MERA simulations. Both the noisy simulation (shaded area) and the experimental data (dots) are shown with indicators for the 95% confidence intervals. The results are based on the same dataset as Fig. 3, but here, positive semi-definite density matrices are reconstructed using maximum-likelihood estimation.

Figure 4a and d show the two lowest entanglement eigenvalues {*ζ*_0_, *ζ*_1_} of ${\hat{\varrho }}_{B}$ for the TFIM (1), revealing distinct behaviors at and away from the critical point. At criticality (*g* = 1), the lowest eigenvalue *ζ*_0_ exhibits a log-law scaling like the entanglement entropy, while *ζ*_1_ decreases with increasing *T*. As shown in Fig. 4b, the Schmidt gap *Δ*_*λ*_ = *λ*_0_ − *λ*_1_ reduces by approximately 1/24 per renormalization step, which agrees with the approximate theoretical prediction *Δ*_*λ*_ ~ *ℓ*^−1/24^ from ref. ^59^, where *ℓ* denotes again the (effective) subsystem size. For the non-critical point *g* = 1.5, Fig. 4d shows that *ζ*_0_ and *ζ*_1_ saturate with increasing *T*.

Since *g* = 1.5 lies in the ${{\mathbb{Z}}}_{2}$-symmetric paramagnetic phase, all subsystem density matrices of the ground state must commute with the operator ${\otimes }_{i}{\hat{Z}}_{i}$. We verify this symmetry by measuring the spin-flip operator on subsystem *B*. As discussed above, the measurement is done efficiently with the compressed representation of ${\hat{\varrho }}_{B}$ on the renormalized sites, exploiting the fact that, for *g *≥ 1, the unitary ${\hat{U}}_{B}$ which transforms the measured state to the actual state on *B* preserves the ${{\mathbb{Z}}}_{2}$-symmetry. As observed in Fig. 4e, the associated entanglement Hamiltonian exhibits a ground state in the even-parity sector and a first excited state in the odd sector. This mirrors the properties of the system Hamiltonian, aligning with recent efforts concerning connections between entanglement Hamiltonians and system Hamiltonians^17,60^.

(iv) Subsystem fidelity: Finally, we compare the reconstructed subsystem density matrix ${\hat{\varrho }}_{B}$ in the ${\hat{U}}_{B}$ basis with the ideal MERA simulations, and show the infidelity $1-{{{\mathcal{F}}}}$ in Fig. 4c,f for the transverse fields *g* = 1.0 and 1.5, where the fidelity is defined as

3 $${{{\mathcal{F}}}}={\left({{{\rm{Tr}}}}\sqrt{\sqrt{{\hat{\varrho }}_{B}}{\hat{\rho }}_{{{{\rm{ideal}}}}}\sqrt{{\hat{\varrho }}_{B}}}\right)}^{2}.$$

We achieve infidelities ranging from 10^−4^ to 10^−2^, which enables the clear discrimination of different entanglement scaling laws, even when the absolute difference in *S* is small. These high fidelities arise from careful calibrations, optimized ion-qubit mappings, the intrinsic noise resilience of MERA, suitable gate decompositions for the MERA circuit, and the efficient holographic tomography method.

**Fig. 5:**
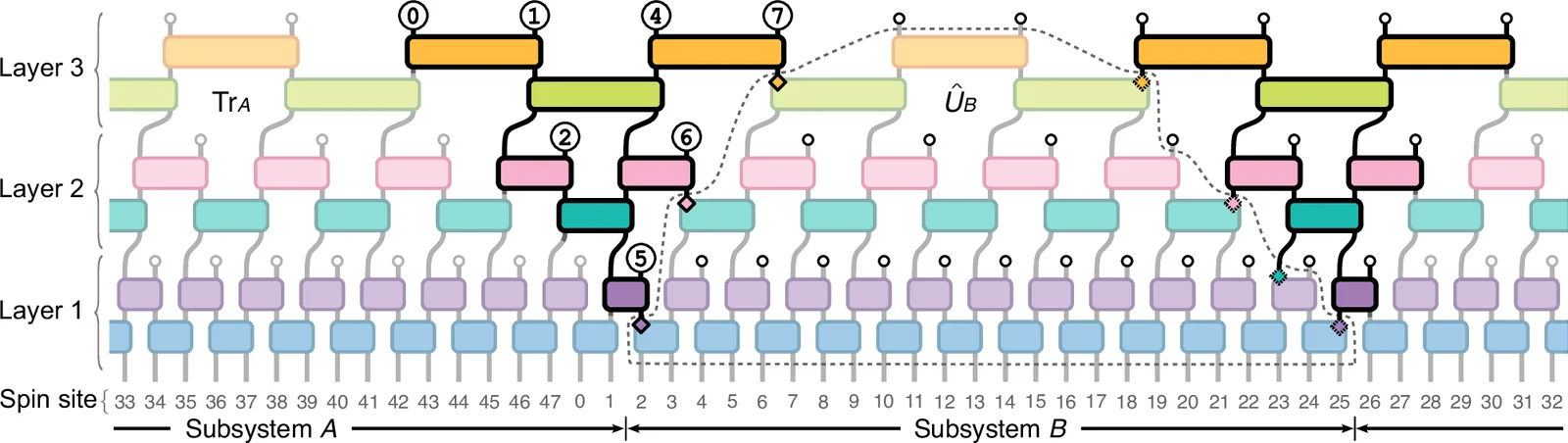
Holographic subsystem tomography and entanglement scaling. An infinite binary MERA with *T* layers is consistent with a MERA on 6 × 2^*T*^ sites with periodic boundary conditions. Here, *T* = 3, resulting in a total of 6 × 2^3^ = 48 sites. All connected two-point correlation functions for sites of distance ≥3 × 2^*T*^ − 2 = 22 are exactly zero. For example, $\langle {\hat{X}}_{10}{\hat{Y}}_{31}\rangle -\langle {\hat{X}}_{10}\rangle \langle {\hat{Y}}_{31}\rangle$ can be nonzero while $\langle {\hat{X}}_{10}{\hat{Y}}_{32}\rangle -\langle {\hat{X}}_{10}\rangle \langle {\hat{Y}}_{32}\rangle \equiv 0$. This is due to the fact that, in the latter case, the causal cones of the two operators have no overlap. Similarly, for any bipartition of the system into equally sized blocks like *A* = {26, …, 47, 0, 1} and *B* = {2, …, 25}, the causal cones of the two subsystem boundaries do not overlap. Hence, measurements of the renormalized sites at the two boundaries are independent of each other, i.e., follow a product distribution.

Recall that we determine ${\hat{\varrho }}_{B}$ through tomography on only ~*T* renormalized sites at the edge of subsystem *A*’s causal cone (also see Fig. 5), where, in the basis defined by ${\hat{U}}_{B}$, all other qubits are in the $\left\vert 0\right\rangle$ state and need not be implemented. This is sufficient because the fidelity metric (3) and bipartite entanglement properties are invariant under the unitary transformation ${\hat{U}}_{B}$.

Also, while many widely used variational ansätze employ CNOT gates, our circuits use two-qubit *XX* Mølmer-Sørensen gates with typical absolute rotation angles in the range from 0.01*π* to 0.2*π*, which are much smaller than those of fully entangling gates. As Mølmer-Sørensen gate errors decrease with the rotation angle, this leads to significantly reduced experimental errors. Supplementary Note 6 provides a breakdown of error contributions and explains how the infidelity scales with experimental errors.

## Discussion

We employed MERA on an ion-trap quantum computer to clearly resolve the quantum phase transition in a condensed matter system, successfully capturing the universal properties of Ising-class physics. Furthermore, our holographic subsystem tomography method made it possible to demonstrate the transition from area-law to log-area-law scaling in ground-state entanglement entropies. This entanglement scaling transition is a hallmark of quantum many-body physics with no classical analog. Its experimental observation had previously faced substantial challenges, including finite-size effects and attainable fidelities.

The advantages of the MERA approach become particularly clear when compared to matrix product states (MPS). To capture critical ground states, the sequential geometry of an MPS requires circuit depths scaling as ${{{\mathcal{O}}}}(L)$ and a polynomially growing bond dimension. In contrast, MERA achieves this with a logarithmic depth ${{{\mathcal{O}}}}(\log L)$ and a constant bond dimension owing to its unique hierarchical structure. Finally, while MPS approaches strongly favor 1D systems, MERA captures area-law states across all spatial dimensions *D* with size-independent bond dimensions^61,62^.

The investigation of more complicated models, including systems with higher spin and spatial dimensions, generally requires larger MERA bond dimensions *χ* ≳ 4. As an example, Supplementary Note 3 discusses numerical simulations of Trotterized MERA circuits^25,26^ with *χ* = 4 to demonstrate the Berezinskii-Kosterlitz-Thouless (BKT) phase transition in XXZ spin chains. In classical MERA simulations, the primary bottleneck is tensor contractions, whose cost scales as ${{{\mathcal{O}}}}({\chi }^{r})$, where the exponent *r* depends on the spatial dimension and specific MERA structure. In 1D with *r* = 7, …, 9, these costs are still tractable for moderate bond dimensions *χ* ≲ 20^22–24^. However, for higher-dimensional systems such as (quasi-)2D frustrated quantum magnets and fermionic systems, where quantum Monte Carlo generally fails due to the negative sign problem, the classical contraction costs with scaling exponents *r* = 16, …, 26 become prohibitive even for small *χ*^24,63^.

To avoid these high contraction costs, our prior work^25,26^ proposed implementing Trotterized MERA on quantum computers. As discussed in refs. ^25,26,28^, the required qubit count grows only logarithmically with *χ* and is independent of system size if mid-circuit resets are available. The circuit depth scales as ${{{\mathcal{O}}}}(Tt)$, where the number of MERA layers $T \sim \log \xi$ governs the maximal correlation length *ξ*, and *t* is a tunable Trotter depth that controls the expressiveness of sub-circuits in individual tensors. For example, simulating a large (e.g., hundreds-by-hundreds) 2D system with a 2 × 2 ↦ 1 MERA using *χ* = 8, *T* = 6, and *t* = 2 requires either (i) 108 resettable qubits and a circuit depth of 96 when minimizing depth or (ii) 42 qubits and depth 624 when minimizing the qubit count. Such circuits are not classically tractable, yet they are realistic for state-of-the-art quantum hardware.

Recent advances in trapped-ion platforms have brought these capabilities closer to realization. For example, 56-qubit systems with ≥99.8% two-qubit gate fidelity are now available^64^, and circuits with over 2000 two-qubit gates have been demonstrated^65^. Larger systems, with  ~ 200 qubits, can be achieved by scaling up the system used in this work^66^. The primary error source in high-connectivity long ion chains—motional heating of low-frequency modes—can be reduced by over two orders of magnitude using cryogenic techniques and sympathetic cooling with a different isotopic species^67^. This approach also facilitates qubit reset and reuse, further enhancing scalability.

Since MERA has been rigorously proven to be free of barren plateaus^30,31^, its variational optimization using quantum computers is feasible. More importantly, optimizing MERA with the aid of quantum computers provides a polynomial quantum advantage over classical MERA optimization, which is particularly pronounced in *D*≥2 spatial dimensions^26^.

Once an optimized MERA state is determined, rich physics can be probed through local observables, correlators, and entanglement measures. Measuring local observables and correlators is straightforward, requiring similar resources as energy estimation. Probing entanglement is more demanding, as large-*χ* MERA involves multiple qubits per renormalized site, making holographic subsystem tomography costly. To address this, one can perform randomized measurements on edges of the boundary causal cones, and adopt the idea of entanglement Hamiltonian tomography^17,60^ to learn the renormalized entanglement Hamiltonian, assuming a structured form in terms of renormalized degrees of freedom.

Beyond the study of strongly correlated many-body ground states, MERA states can serve as high-quality starting points for the investigation of nonequilibrium dynamics. As demonstrated in this work, MERA accurately captures both critical and non-critical ground states. A natural extension is to prepare a MERA state and evolve it under quench or Floquet dynamics. Since entanglement growth under nonequilibrium dynamics generally leads to volume-law scaling of entanglement entropies, a quantum advantage is expected as soon as we surpass the realm of exact classical Krylov-subspace methods for  ≳ 36 qubits.

## Methods

### Holographic subsystem tomography and entanglement scaling

For the study of bipartite entanglement entropies, entanglement spectra, and subsystem state fidelities as discussed in Results, we consider MERA approximations $\left\vert \Psi \right\rangle$ for the ground state and a spatial bipartition into subsystem *A* and its complement *B*.

The goal of the holographic subsystem tomography is to efficiently access the reduced density matrix ${\hat{\varrho }}_{B}={{{{\rm{Tr}}}}}_{A}\vert \Psi \rangle \langle \Psi \vert$ of subsystem *B* in the experiment. The isometric property (${\hat{W}}^{{{\dagger}} }\hat{W}={\mathbb{1}}$) of the MERA tensors implies that, under the partial trace ${{{{\rm{Tr}}}}}_{A}$, all tensors outside the causal cone of *B* can be removed, as exemplified by the shaded area atop subsystem *A* in Fig. 5. Furthermore, the aforementioned quantities are all invariant under unitary transformations on subsystem *B*. Using this invariance, we can remove all MERA gates outside the causal cone of subsystem *A*, which corresponds to the action of the unitary transformation ${\hat{U}}_{B}^{{{\dagger}} }$. Only the gates inside the causal cone(s) of the subsystem boundary(ies) remain. All qubits outside this causal cone are in the reference state $\left\vert 0\right\rangle$ and need not be implemented in the experiment.

To determine the transformed subsystem density matrix

4 $${\hat{U}}_{B}^{{{\dagger}} }{\hat{\varrho }}_{B}{\hat{U}}_{B}={{{{\rm{Tr}}}}}_{A}({\hat{U}}_{B}^{{{\dagger}} }\vert \Psi \rangle \langle \Psi \vert {\hat{U}}_{B}),$$

we only need to measure the ~*T* non-trivial renormalized sites at the edge of *A*’s causal cone, as indicated by the diamonds in Fig. 5. Again *T* denotes the number of MERA layers.

To further reduce experimental resource requirements, we consider either infinite chains with a bipartition into two semi-infinite subsystems, or finite chains with periodic boundary conditions where block *B* is large enough such that the causal cones of its two boundaries are disjoint as exemplified in Fig. 5. In the latter scenario, the transformed density operator (4) is actually a tensor product of a state ${\tilde{\varrho }}_{B}^{L}$ at the left edge, a state ${\tilde{\varrho }}_{B}^{R}$ at the right edge, and central sites in the reference state $\vert 0\rangle \langle 0\vert$,

5 $${\hat{U}}_{B}^{{{\dagger}} }{\hat{\varrho }}_{B}{\hat{U}}_{B}={\tilde{\varrho }}_{B}^{L}\otimes \vert 0\rangle \langle 0\vert \otimes \ldots \otimes \vert 0\rangle \langle 0\vert \otimes {\tilde{\varrho }}_{B}^{R}.$$

Due to the product structure, ${\tilde{\varrho }}_{B}^{L}$ and ${\tilde{\varrho }}_{B}^{R}$ can be measured independently.

Moreover, when studying quantities that only depend on the spectrum of ${\hat{\varrho }}_{B}$ for a bipartition of the system into two equally sized halves and for total system sizes *L* = 6 × 2^*T*^, 8 × 2^*T*^, ..., it is sufficient to measure only one of the two density operators. In this case, the MERA circuits for ${\tilde{\varrho }}_{B}^{L}$ and ${\tilde{\varrho }}_{B}^{R}$ prepare the same pure state, and only differ in the selection of sites to be measured: those at the right edge of the causal cone for ${\tilde{\varrho }}_{B}^{L}$ and those at the left edge for ${\tilde{\varrho }}_{B}^{R}$, as indicated in Fig. 5. Thus, all nonzero eigenvalues of ${\hat{\varrho }}_{B}$ are given by the elementwise product

6 $$\{{\lambda }_{i}{\lambda }_{j}\} \, {{{\mbox{of}}}} \, {{\tilde{\varrho }}_{B}^{L}}{\hbox{'}} {\mbox{s}} \, {\mbox{spectrum}} \, \{{\lambda }_{i}\}$$

with itself.

### Subsystem size versus number of MERA layers

In the main text, we associated the number *T* of layers in infinite MERA with the logarithm of subsystem sizes *ℓ*. Here, we elaborate further on this relation.

An infinite binary 1D MERA $\left\vert \Psi \right\rangle$ with *T* layers has a maximum correlation range *ξ*_max_ ≔ 3 × 2^*T*^, where the prefactor 3 arises from the maximal causal-cone width of local operators acting on *k *≤ 3 contiguous sites. In particular, connected correlation functions

7 $${\langle {\hat{O}}_{i}{\hat{O}}_{i+\delta \ell }^{{\prime} }\rangle }_{\Psi }-{\langle {\hat{O}}_{i}\rangle }_{\Psi }{\langle {\hat{O}}_{i+\delta \ell }^{{\prime} }\rangle }_{\Psi }$$

are exactly zero for all *i* and local operators ${\hat{O}}_{i}$ and ${\hat{O}}_{j}^{{\prime} }$ whenever *δℓ*≥*ξ*_max_ − 3 + *k*.

Hence, all local observables, two-point correlation functions, and subsystem density matrices for blocks of *ℓ *≤ *ξ*_max_ sites of an infinite MERA coincide with those of a corresponding finite-size MERA for a system of 2*ξ*_max_ = 6 × 2^*T*^ sites with periodic boundary conditions.

Furthermore, the half-chain density operator of an infinite *T*-layer MERA, considered in Results, is given by

8 $${\hat{U}}_{B}^{{{\dagger}} }{\hat{\varrho }}_{B}{\hat{U}}_{B}={\tilde{\varrho }}_{B}^{L}\otimes \vert 0\rangle \langle 0\vert \otimes \vert 0\rangle \langle 0\vert \otimes \ldots,$$

where ${\tilde{\varrho }}_{B}^{L}$ is the same state as the one in Eq. (5) for an *ℓ* = 3 × 2^*T*^-site block in a periodic 6 × 2^*T*^-site system, establishing the exponential relation between *ℓ* and *T*. The spectra of the states (8) and (5) are related by the elementwise product (6).

### Scale-invariant MERA

**Fig. 6:**
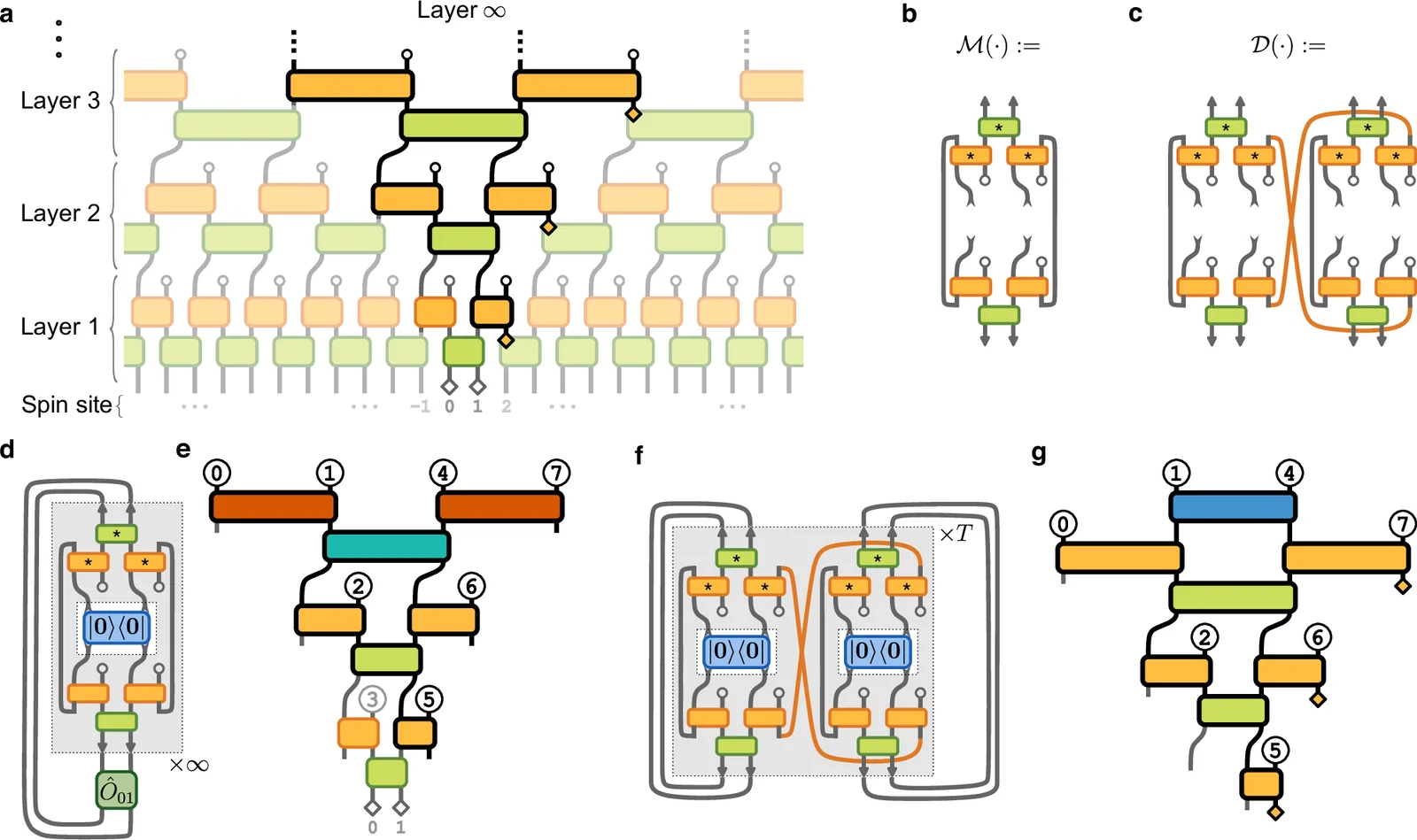
Scale-invariant MERA and its implementation on the quantum computer. **a** A scale-invariant binary 1D MERA with an infinite number of lattice sites and layers. All unitaries (bare rectangles) and isometries (rectangles with attached circles) are identical across layers and the spatial direction. **b** Local observables on sites 0 and 1 have a causal cone consisting of a repeating configuration of tensors in each layer. The corresponding repeated layer-transition channel ${{{\mathcal{M}}}}(\cdot )$ maps reduced density matrices from the top to the bottom layer. Stars indicate complex conjugation. **c** The shown doubled and SWAP-contracted layer-transition map ${{{\mathcal{D}}}}$ can be used to compute the second-order Rényi entanglement entropy of a bipartition into subsystems (−*∞*, 1] and [2, *∞*) (see panel (**f**)). **d** Causal-cone tensor-network for the evaluation of local expectation values on sites 0 and 1. The MERA circuit is initialized with reference state $\left\vert 0\right\rangle$ at the top layer. **e** In the experiment, we truncate the scale-invariant MERA after layer *T*. For the measurement of local observables, the top layer is optimized to approximate the dominant eigenmode ${\hat{\rho }}_{{{{\rm{steady}}}}} : \!\!={{\lim }_{T\to \infty }}{{{{\mathcal{M}}}}}^{T}(\vert {{{\bf{0}}}}\rangle \langle {{{\bf{0}}}}\vert )$ of the layer-transition channel. Finally, we measure the qubits at sites 0 and 1. **f** The tensor network for the purity of the reduced density operator of subsystem [2, *∞*). **g** In this case, the top tensor (blue) is optimized to suppress sub-leading contributions from the eigenvectors of the doubled map ${{{\mathcal{D}}}}$. Scale-invariant layers are then appended. To extract the scaling of entanglement as a function of the number *T* of RG steps, we perform tomography on *T* qubits at the right edge of the boundary causal-cone circuit (see also Fig. 5).

Scale-invariant MERA are designed for critical systems, where correlation lengths diverge. As shown in Fig. 6a, they feature an elementary cell of tensors that repeats in the spatial and renormalization directions, in accordance with the self-similarity of critical systems.

Viewed in the renormalization direction, the scale-invariant MERA maps the local Hamiltonian of a critical system into an RG fixed point. Seen in reverse, the scale-invariant MERA acts as a quantum channel that iteratively refines the state, starting from an initial reference state and adding layers to construct the ground state of the critical system.

Consider spin sites 0 and 1. Their causal-cone state, as indicated by the unshaded region in Fig. 6a, is constructed by applying the same layer-transition channel ${{{\mathcal{M}}}}$ (Fig. 6b) multiple times, where renormalized sites that leave the causal cone are traced out. ${{{\mathcal{M}}}}$ is a quantum channel and can be diagonalized in bi-orthonormal operator bases such that

9 $${{{\mathcal{M}}}}(\cdot )= \vert {\hat{\varrho }}_{{{{\rm{steady}}}}} \rangle \rangle \langle \langle {\mathbb{1}} \vert+ {\sum}_{i > 0}{m}_{i} \vert {\hat{r}}_{i} \rangle \rangle \langle \langle {\hat{\ell }}_{i} \vert,$$

where ∣*m*_*i*_∣ < 1 for all *i* > 0, and we use a super-bra-ket notation for the operator-basis elements according to the Hilbert-Schmidt inner product $\langle \langle \hat{L}| \hat{R}\rangle \rangle : \!\!={{{\rm{Tr}}}}({\hat{L}}^{{{\dagger}}}\hat{R})$. With ${\hat{r}}_{0}\equiv {\hat{\varrho }}_{{{{\rm{steady}}}}}$ and ${\hat{\ell }}_{0}\equiv {\mathbb{1}}$, ${\hat{r}}_{i}$ and ${\hat{\ell }}_{i}$ are the right and left eigen-operators of ${{{\mathcal{M}}}}$ for eigenvalue *m*_*i*_ and obey $\langle \langle {\hat{\ell }}_{i}| {\hat{r}}_{j}\rangle \rangle={\delta }_{i,j}$.

We initialize the MERA in the product state $\left\vert {{{\bf{0}}}}\right\rangle=\left\vert 0,\ldots,0\right\rangle$ at the topmost (infinite) layer. The expectation value of a local observable ${\hat{O}}_{0,1}$ with the scale-invariant MERA then is

10 $${{\lim }_{T\to \infty }}{{{\rm{Tr}}}}\left({\hat{O}}_{0,1}{{{{\mathcal{M}}}}}^{T}(\vert {{{\bf{0}}}}\rangle \langle {{{\bf{0}}}}\vert )\right)={{{\rm{Tr}}}}({\hat{O}}_{0,1}{\hat{\varrho }}_{{{{\rm{steady}}}}}).$$

See Fig. 6d. Experimentally, we cannot directly implement MERA with an infinite number of layers. Instead, we truncate after layer *T* and use the top layer to prepare a two-site state ${\hat{\varrho }}^{{\prime} }$ that maximizes the contribution of the dominant eigenmode ${\hat{\varrho }}_{{{{\rm{steady}}}}}$ of the layer-transition channel (9). This is achieved by choosing the gate angles {**θ**} in layer *T* as $\arg {{\min }_{{{{\mathbf{\theta }}}}}}{\sum }_{i\ > \ 0}| {m}_{i}\langle \langle {\hat{\ell }}_{i}| {\hat{\varrho }}^{{\prime} }\rangle \rangle {| }^{2}$. We then append *T* − 1 layers of the scale-invariant MERA to refine the dominant eigenmode as shown in Fig. 6e. This approach is highly effective as demonstrated for *T* = 5 and a few local observables in Results.

To account for layer truncations in the study of subsystem purity and entanglement, we introduce a doubled SWAP-contracted layer-transition map ${{{\mathcal{D}}}}$ as shown in Fig. 6c. For a scale-invariant MERA truncated after layer *T* and initialized with the reference state $\left\vert {{{\bf{0}}}}\right\rangle$, the purity of subsystem *B* = [2, *∞*) can be computed by iteratively applying ${{{\mathcal{D}}}}$ to the (doubled) zero-product reference state $\vert {{{\bf{0}}}},{{{\bf{0}}}}\rangle \langle {{{\bf{0}}}},{{{\bf{0}}}}\vert$ at the topmost layer and taking the final trace as

11 $${{{\rm{Tr}}}}({\hat{\varrho }}_{B}^{2})={{{\rm{Tr}}}}\left({{{{\mathcal{D}}}}}^{T}(\vert {{{\bf{0}}}},{{{\bf{0}}}}\rangle \langle {{{\bf{0}}}},{{{\bf{0}}}}\vert )\right);$$

see Fig. 6f. Similarly to the channel ${{{\mathcal{M}}}}$, we can diagonalize ${{{\mathcal{D}}}}$ such that ${{{\mathcal{D}}}}(\cdot )={\sum }_{i}{d}_{i}\vert {\hat{R}}_{i}\rangle \rangle \langle \langle {\hat{L}}_{i}\vert$ with ∣*d*_0_∣ > ∣*d*_*i*_∣ for all *i* > 1. Due to the SWAP operation, ${{{\mathcal{D}}}}$ is not a quantum channel and ∣*d*_0_∣ < 1. The second-order Rényi entanglement entropy with *α* = 2 in Eq. (2) evaluates to

12 $${S}_{B}^{(2)}=	-{\log }_{2}\left({{{\rm{Tr}}}}\left({{{{\mathcal{D}}}}}^{T}(\vert {{{\bf{0}}}},{{{\bf{0}}}}\rangle \langle {{{\bf{0}}}},{{{\bf{0}}}}\vert )\right)\right) \\=	-{\log }_{2}\left({\sum}_{i=0}{d}_{i}^{T}\langle \langle {\mathbb{1}}| {\hat{R}}_{i}\rangle \rangle \langle \langle {\hat{L}}_{i}| {{{\bf{0}}}},{{{\bf{0}}}}\rangle \rangle \right) \\ \to 	-T{\log}_{2}{d}_{0}-{\log }_{2}\left(\langle \langle {\mathbb{1}}| {\hat{R}}_{0}\rangle \rangle \langle \langle {\hat{L}}_{0}| {{{\bf{0}}}},{{{\bf{0}}}}\rangle \rangle \right),$$

where the large-*T* limit is taken in the last line, and $\left.\left\vert {{{\bf{0}}}},{{{\bf{0}}}}\right\rangle \right\rangle$ represents the projector $\vert {{{\bf{0}}}},{{{\bf{0}}}}\rangle \langle {{{\bf{0}}}},{{{\bf{0}}}}\vert$. Hence, the entanglement entropy should increase linearly with *T*, i.e., by a constant in each renormalization step.

Even with the small bond dimension *χ* = 2, the dominant eigenvalue *d*_0_ of ${{{\mathcal{D}}}}$ in the optimized scale invariant MERA agrees well with the conformal field theory prediction for the Ising model with central charge *c* = 1/2:

13 $$-8{\log }_{2}{d}_{0}=0.475\ldots \approx c.$$

Again, we truncate the scale-invariant MERA after layer *T*≤4 for experiments. To mitigate the effect of the truncation, we do not initialize with the reference state $\left\vert {{{\bf{0}}}}\right\rangle$ at the top layer but with a more suitable two-site state $\left\vert \varphi \right\rangle$, generated from $\left\vert {{{\bf{0}}}}\right\rangle$ through one entangling and further single-qubit gates. These gates are chosen to suppress subleading contributions in (12) by minimizing ${\sum }_{i\ > \ 0}| {d}_{i}\langle \langle {\mathbb{1}}| {\hat{R}}_{i}\rangle \rangle \langle \langle {\hat{L}}_{i}| \varphi, \varphi \rangle \rangle {| }^{2}$. Thus, we arrive at the approximation

14 $${S}_{B}^{(2)}=-{\log}_{2}({{{\rm{Tr}}}}({{{{\mathcal{D}}}}}^{T}(\vert \varphi , \varphi \rangle \langle \varphi ,\varphi \vert )))\approx -T{\log }_{2}{d}_{0}.$$

After implementing such *T*-layer approximations of a scale-invariant MERA in the experiment, we perform the holographic subsystem tomography, measuring all *T* qubits at the right edge of the boundary causal cone as discussed in Results and illustrated in Fig. 6g.

### System characterization and error model

(i) State preparation and measurement (SPAM) error: SPAM errors contribute significantly to our error budget. To quantify the probability *p*_*d*_ of SPAM errors for the dark state ($\left\vert 0\right\rangle$), we performed fluorescence detection after initializing all qubits via the usual optical pumping, yielding an average measured error of 0.16%. To measure the probability *p*_*b*_ of SPAM errors for the bright state ($\left\vert 1\right\rangle$), we applied *R*_*y*_(*π*) gates following the optical pumping and then performed fluorescence detection, yielding an average measured error of 0.45%. Further details and run-by-run results are provided in the Supplementary Note 4.

**Fig. 7:**
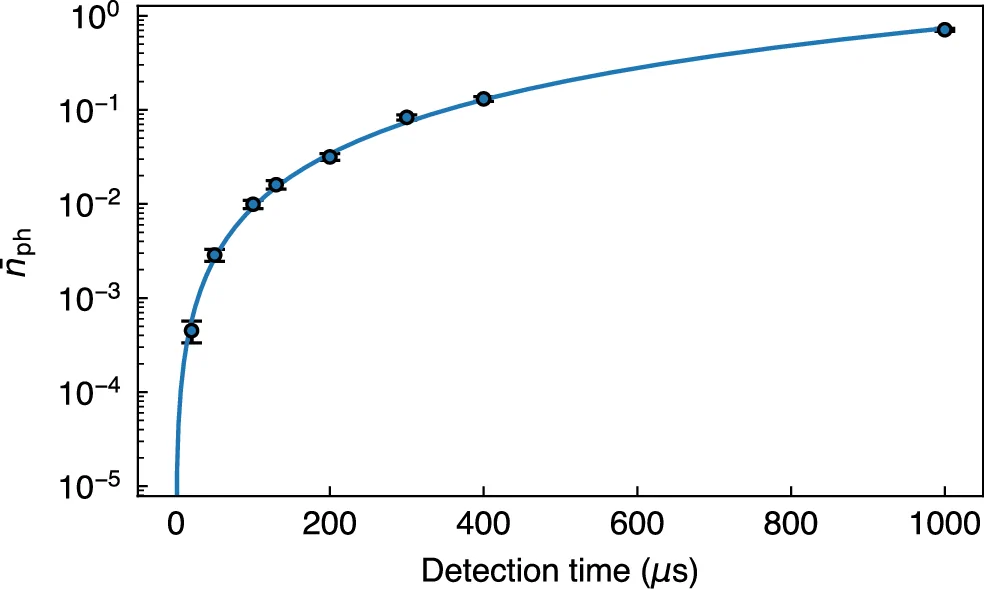
Off-resonant pumping from the dark to the bright state during detection. The average number ${\bar{n}}_{{{{\rm{ph}}}}}$ of collected photons when preparing the ion in the $\left\vert 0\right\rangle$ is plotted as a function of detection time *τ*. The error bars indicate a 1*σ* confidence interval with 60,000 experimental shots.

We expect our SPAM errors to be dominated by off-resonant optical pumping during the detection pulse^68,69^, with an additional preparation error for the $\left\vert 0\right\rangle$ state. To distinguish the preparation error from the measurement error, we fitted the average dark-state photon number as a function of detection time *τ* (Fig. 7) to the rate equation model from ref. ^70^:

15 $${\bar{n}}_{{{{\rm{ph}}}}}=\int_{0}^{\tau} \eta {{{\Gamma}}}_{{{{\rm{sc}}}}}\left({P}_{d,\infty}-(p-{P}_{b,\infty})\,{{{{\rm{e}}}}}^{-({R}_{b}+{R}_{d})t}\right){{{\rm{d}}}}t,$$

where *p* is the dark-state preparation error probability. Here, ${P}_{b,\infty }={({R}_{b}/{R}_{d}+1)}^{-1}$ and *P*_*d*,*∞*_ = 1 − *P*_*b*,*∞*_ are the equilibrium bright and dark-state populations, respectively. The pumping rates *R*_*b*_ = 300(1)/*s* and *R*_*d*_ = 18(1)/*s* were determined by fitting count histograms as described in the Supplementary Note 4. The fitted value *p* = 10(6) × 10^−5^ confirms that our SPAM error is dominated by the measurement errors, as *p* ≪ *p*_*d*_, *p*_*b*_.

To model state-preparation error in the numerical simulations, we flip each qubit with probability *p*. To simulate the detection error, after applying the circuit and measuring in the computational basis $\{\left\vert 0\right\rangle,\left\vert 1\right\rangle \}$, we randomly flip $\left\vert 0\right\rangle \to \left\vert 1\right\rangle$ with probability *p*_*d*_ − *p* and $\left\vert 1\right\rangle \to \left\vert 0\right\rangle$ with probability *p*_*b*_ − *p*.

(ii) Idling noise: We characterized the dephasing of idle qubits using a Ramsey pulse sequence. After applying ${R}_{y}(\frac{\pi }{2})$ gates to all 13 qubits, we simulated idling by turning off the individual-addressing Raman beams while keeping the global beam on with the same settings as during a gate sequence. After a variable idle time *τ*_idle_, we completed the Ramsey sequence with *R*_*z*_(*ϕ*) and ${R}_{y}(\frac{\pi }{2})$ gates and recorded the $\left\vert 1\right\rangle$ state population as a function of *ϕ*. We obtained the fitted ion-averaged contrast *C* from the resulting fringes and modeled it as $A\,{{{{\rm{e}}}}}^{-{\tau }_{{{{\rm{idle}}}}}/{T}_{2}^{*}}$. From several datasets collected on different experimental days, the fitted ${T}_{2}^{*}$ ranges from 260 ms to 370 ms, with an average of 290 ms. In Supplementary Note 5, there is a representative fit from a sample dataset, yielding *A* = 0.991(1) and ${T}_{2}^{*}=300(17)$ ms. We attribute the measured dephasing to acoustic noise, mainly from air cooling fans, in our phase-sensitive Raman interferometer setup. We expect that ${T}_{2}^{*}$ times of several seconds can be achieved using phase-insensitive schemes^71^ that are compatible with our experimental setup.

In our simulations, we model the effects of dephasing on the qubit density matrix $\hat{\rho }$ over the time interval *t* via the quantum channel

16 $${{{{\mathcal{E}}}}}_{Z}(\hat{\rho })=(1-{p}_{Z})\hat{\rho }+{p}_{Z}\hat{Z}\hat{\rho }\hat{Z},$$

with the *Z*-flip probability ${p}_{Z}=t/(2{T}_{2}^{*})$.

(iii) Gate errors: While we actively calibrated the rotation angles, blue-and-red sideband imbalances, and Stark shifts of two-qubit gates before running circuits, residual two-qubit errors still remain and are discussed below.

(iii.a) Axial motion: Axial motion of our 15-ion chain, dominated by the lowest-frequency axial mode (mode 0) with angular frequency *ω*_0_ = 2*π* × 241.8 kHz, is a leading error source in our platform. This motion misaligns the ions relative to the tightly-focused addressing beams, causing gate errors^67^. These errors increase over time as the ions’ motion is driven by noisy electric fields.

**Fig. 8:**
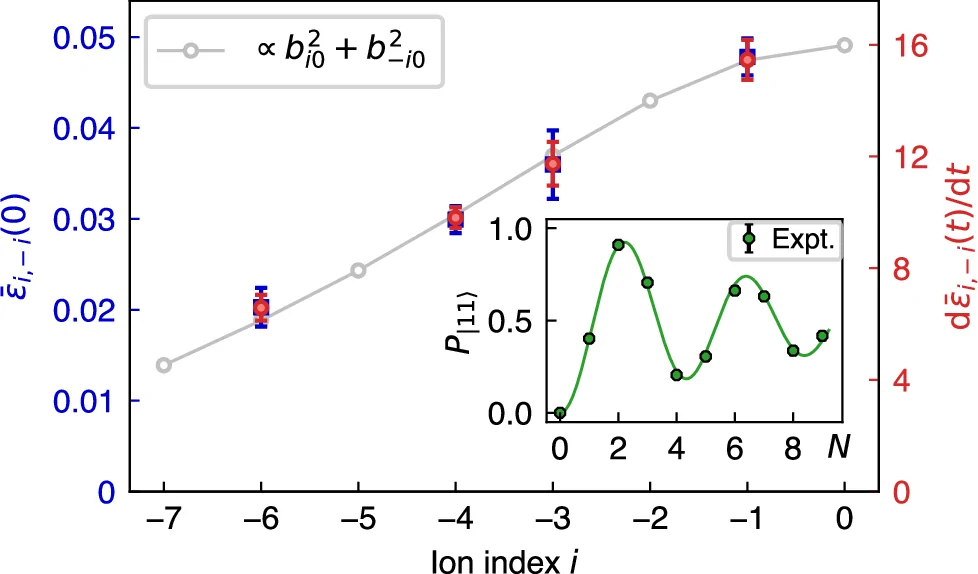
Gate errors due to axial motion. The fitted initial decay parameter ${\bar{\varepsilon }}_{i,-i}(0)$ (blue) and its increase rate ${{{\rm{d}}}}{\bar{\varepsilon }}_{i,-i}(t)/{{{\rm{d}}}}t$ (red) are shown for *XX* gates acting on ions *i* and −*i*. The gray line is proportional to the sum of squares ${b}_{i0}^{2}+{b}_{-i0}^{2}$ of the participation factors for the addressed ions in the lowest axial mode of the ion chain. Error bars indicate 1*σ* fit uncertainties. The inset shows the $\left\vert 1,1\right\rangle$-state population for ions (−3, 3) as a function of the gate number *N* after a wait time of *t* ≈ 10 ms, with a fit according to Eq. (18). The 1*σ* statistical uncertainties (error bars) are smaller than the marker size.

We quantified the addressing error after a wait time *t* following laser cooling by applying a variable number *N* of $XX(\frac{\pi }{2})$ gates of total duration *Δt* to ions *i* and *j* and measuring the mean population ${P}_{\left\vert 11\right\rangle }$ of the $\left\vert 1,1\right\rangle$ state. The recorded populations ${P}_{\left\vert 11\right\rangle }$ as a function of *N* for (*i*, *j*) = (3, −3) are shown in the inset of Fig. 8. Uncertainty in the ions’ axial motion translates into a variance in the total *XX* rotation angle, leading to damping of ${P}_{\left\vert 11\right\rangle }$ oscillations.

The observed damping is governed by the statistics of the phonon number of mode 0 during gate application^67^. We model the evolution of the phonon number *n*_*t*_ at time *t* as a biased random walk. Since the energy of mode 0 following laser cooling corresponds to hundreds of phonons, and the average heating per *XX* gate corresponds to more than 20 phonons, we coarse-grain *n*_*t*_ on intervals of size *δn* with 1≤*δn* ≪ 2*n*_*t*_ and model the random walk as

17 $${n}_{t+\delta t}:=\left\{\begin{array}{ll} {n}_{t}+\delta n\quad &\,{{\mbox{with\,prob.}}}\,\frac{{n}_{t}+\delta n}{\delta n}\frac{\dot{\bar{n}}\delta t}{\delta n}\\ {n}_{t}-\delta n\quad &\,{{\mbox{with\,prob.}}}\,\frac{{n}_{t}}{\delta n}\frac{\dot{\bar{n}}\delta t}{\delta n} \hfill \\ {n}_{t}\quad \hfill &\,{{\mbox{otherwise}}},\hfill\end{array}\right.$$

where $\dot{\bar{n}}$ is the heating rate of mode 0, and the time step *δt* is chosen so that the change of *n*_*t*_/*δn* remains small ($\frac{2{n}_{t}+\delta n}{\delta n}\frac{\dot{\bar{n}}\delta t}{\delta n}\le 1$).

When initialized with a Boltzmann distribution with mean ${\bar{n}}_{0}\gg 1$, *n*_*t*_ remains exponentially distributed with mean ${\bar{n}}_{t}={\bar{n}}_{0}+\dot{\bar{n}}t$. In this thermal limit, the time-averaged phonon number ${n}_{t}^{{\mbox{avg}}\,}: \!\!=\frac{1}{\Delta t}\int_{t}^{t \!+\! \Delta t}{n}_{{t}^{{\prime} }}\,{{{\rm{d}}}}{t}^{{\prime} }$ between time *t* and *t* + *Δt* has the mean ${\mu }_{t}={\bar{n}}_{t+\Delta t/2}={\bar{n}}_{t}+\dot{\bar{n}}\frac{\Delta t}{2}$ and the variance ${\sigma }_{t}^{2}={\mu }_{t}^{2}-\frac{{\bar{n}}_{t}\dot{\bar{n}}\Delta t}{3}+\frac{{\dot{\bar{n}}}^{2}\Delta {t}^{2}}{6}$. The distribution of ${n}_{t}^{{\mbox{avg}}\,}$ can be well approximated by a Gamma distribution with the corresponding mean and variance.

If *N* *XX*(*θ*) gates are applied between time *t* and *t* + *Δt*, neglecting other errors and assuming the above Gamma distribution, we obtain the population

18 $${P}_{\left\vert 11\right\rangle }(t)=\frac{1-C\cos (N\theta -\phi )}{2},$$

of the $\left\vert 1,1\right\rangle$ state with phase shift $\phi=\alpha \arctan (\alpha N\theta {\bar{\varepsilon }}_{i,j}(t+\frac{\Delta t}{2}))$ and contrast $C={(1+{[\alpha N\theta {\bar{\varepsilon }}_{i,j}(t+\frac{\Delta t}{2})]}^{2})}^{-\alpha /2}$, where $\alpha={\mu }_{t}^{2}/{\sigma }_{t}^{2}$ and ${\bar{\varepsilon }}_{i,j}(t)={\bar{\varepsilon }}_{i}(t)+{\bar{\varepsilon }}_{j}(t)$. The mean dimensionless decay parameter ${\bar{\varepsilon }}_{i}(t)$ during the gate sequence is

19 $${\bar{\varepsilon }}_{i}(t)=\frac{\hslash {b}_{i0}^{2}}{m{\omega }_{0}{w}^{2}}{\bar{n}}_{t},$$

where *b*_*i*0_ is the participation factor of ion *i* in mode 0. Here, we assume that the ion motion remains much smaller than our Gaussian-shaped beams. We determine the individual-beam waist *w* by fitting the dependence of the single-qubit Rabi frequency Ω on the axial ion position *x* to ${{{\Omega}}}_{0}{e}^{-{(x-{x}_{0})}^{2}/{w}^{2}}$, yielding *w* = 646(12) nm. In the limit of negligible heating during the gate sequence, the prediction (18) reduces to Eq. (4) in^67^.

For different ion pairs (*i*, *j* = −*i*), we fit the measured mean $\left\vert 1,1\right\rangle$-state population as a function of the gate number *N* and wait time *t* to the model (18). The fitted dimensionless effective decay parameter ${\bar{\varepsilon }}_{i,j}(0)$ at *t* = 0 and its rate of increase ${{{\rm{d}}}}{\bar{\varepsilon }}_{i,j}/{{{\rm{d}}}}t$ are shown as functions of *i* in Fig. 8. Both parameters scale with ${b}_{i0}^{2}+{b}_{j0}^{2}$, indicating that addressing errors are dominated by excitations of the lowest axial mode of the ion chain. The fitted initial phonon number in this mode is ${\bar{n}}_{0}=409(4)$ and the heating rate is $\dot{\bar{n}}=133(1)$ phonons/ms.

To model gate rotation errors for arbitrary single- and two-qubit gates, in each shot of the noisy simulations, we sample the initial phonon number *n*_0_ from an exponential distribution with mean ${\bar{n}}_{0}$. We then evolve *n*_*t*_ using the random walk model (17). For each native gate in the circuit, we compute the time-averaged phonon number ${n}_{t}^{{\mbox{avg}}\,}$. In the noise model, the phonon number ${n}_{t}^{{\mbox{avg}}\,}$ changes single-qubit gate rotations as $R(\theta )\to R(\theta (1-{n}_{t}^{{\mbox{avg}}\,}{\bar{\varepsilon }}_{i}/{\bar{n}}_{t}))$ and two-qubit gate rotations as $XX(\theta )\to XX(\theta (1-{n}_{t}^{{\mbox{avg}}\,}{\bar{\varepsilon }}_{i,j}/{\bar{n}}_{t}))$.

(iii.b) *X*-errors: We describe *X*-errors during an *XX*(*θ*) gate on ion pair (*i*, *j*) using a bit-flip channel acting on the density matrix $\hat{\rho }$ of ion *i* as

20 $${{{{\mathcal{E}}}}}_{X}(\hat{\rho })=(1-{p}_{X})\hat{\rho }+{p}_{X}\hat{X}\hat{\rho }\hat{X},$$

where *p*_*X*_ ≡ *p*_*X*,*i*,*j*_(*θ*) is the *X*-flip probability per gate.

*X*-errors can be caused by residual spin-motion entanglement at the end of Mølmer-Sørensen gates. They can be minimized by carefully choosing the motional detuning, gate duration, and envelope of the amplitude-modulated gate waveforms^72^. In-between experiment runs, we stabilized frequencies of the radial ion-motion modes via blue-sideband Ramsey spectroscopy. Each of the central 11 qubits was used to query one of 11 radial modes that are addressed by the entangling gate waveforms. The obtained spectroscopical data were used to automatically adjust the quadratic and quartic terms in the static axial potential of the trap and a common motional offset frequency for all gates.

**Fig. 9:**
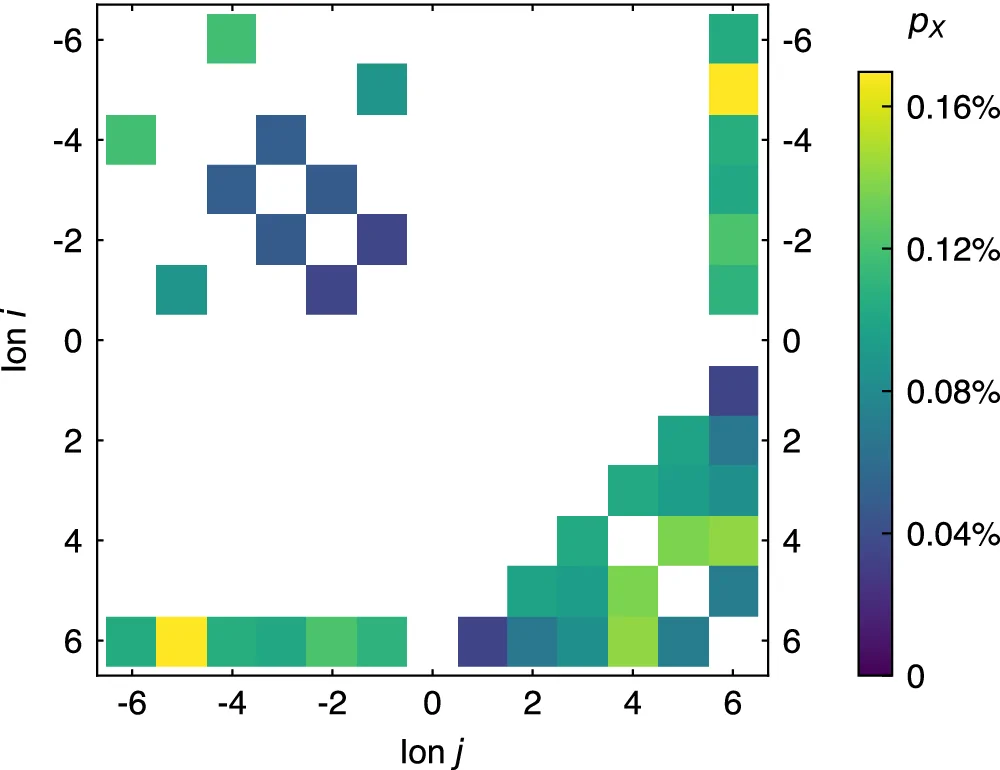
Pauli *X* error per fully-entangling *XX* gate. The Pauli *X* error *p*_*X*,*i*,*j*_(*π*/2) for gates acting on ions *i* and *j* is calculated using Eq. (21). We only show error data for gates that are actually utilized in this work.

After applying *N* consecutive $XX(\frac{\pi }{2})$ gates to the $\left\vert 0,0\right\rangle$ state on ions *i* and *j*, we measured the probability of *X*-flip errors by tracking the population *P*_*i*,*j*_(*N*) of the $\left\vert 0,1\right\rangle$ and $\left\vert 1,0\right\rangle$ states (parity leakage). We distinguished *X*-flip errors from coherent *XX* gate crosstalk by focusing on the shots where only the target qubits are flipped. In the noise model (20), we employ the *X*-flip error probabilities

21 $${p}_{X,i,j}(\theta )=\frac{| \theta | }{\pi /2}\frac{{P}_{i,j}(N)}{2N},$$

which are summarized in Fig. 9.

(iii.c) *Z*-errors: To measure Stark shifts during a specific *XX* gate, we insert *N* pairs of $XX(\frac{\pi }{2})-XX(-\frac{\pi }{2})$ gates into a Ramsey pulse sequence consisting of initial ${R}_{y}(\frac{\pi }{2})$ and final ${R}_{z}(\phi )-{R}_{y}(\frac{\pi }{2})$ pulses applied to each targeted ion. After adjusting the qubit frequencies during the gate to compensate for the measured shifts, we use the same sequence to estimate any residual gate *Z*-errors.

**Fig. 10:**
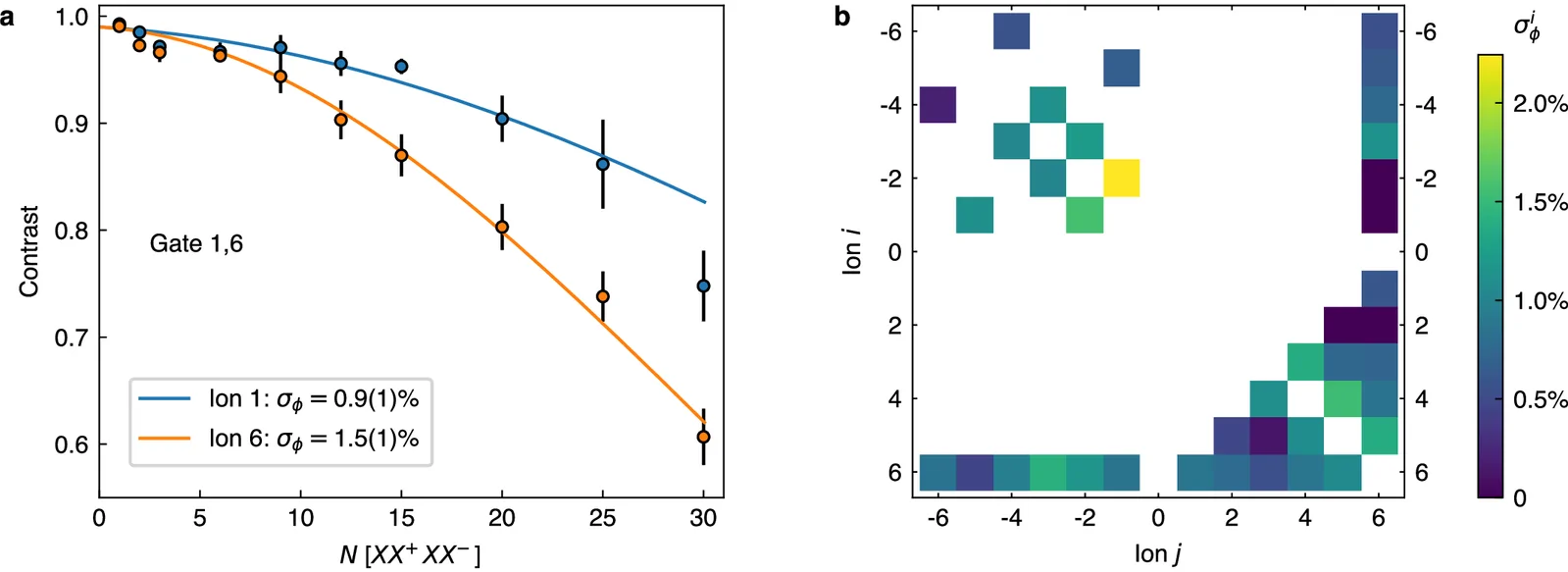
Effect of Stark shifts on *XX* gates. **a** Ramsey fringe contrast as a function of the number of (*XX*^+^, *XX*^−^) gate pairs inside the Ramsey sequence for a sample gate. Error bars indicate 1*σ* fit uncertainties. Solid lines indicate fits to a Gaussian function with zero mean and standard deviation $N{\sigma }_{\phi }^{i}$. **b** Standard deviation ${\sigma }_{\phi }^{i}$ of the random Gaussian-distributed phase shift *ϕ*_*i*_ on ion *i* per fully-entangling *XX* gate acting on ion pair (*i*, *j*). Error data are shown only for gates utilized in this work.

The fitted contrast of the obtained Ramsey fringes for a sample gate is shown in Fig. 10a. The observed dependence of the contrast on *N* is roughly quadratic, indicating that the phase shift error accumulates coherently as the number of gates increases. We model the observed decoherence by correlated fluctuations in the gate-induced Stark shift, which can arise from fluctuations in the amplitude balance of the red and blue tones of the Mølmer-Sørensen gate due to air turbulence in the Raman beam setup. Since our Ramsey sequence prepares the qubits in the *X*-basis, we do not expect gate *X*-errors to affect the measured contrast. We assume that the phase shift *ϕ*_*i*_ induced on ion *i* by a fully-entangling *XX* gate during each shot follows a Gaussian distribution with zero mean and standard deviation ${\sigma }_{\phi }^{i}$. This noise model results in a fringe contrast proportional to ${{{{\rm{e}}}}}^{-{(N{\sigma }_{\phi }^{i})}^{2}/2}$ to first order. Figure 10 shows fits of this model to the measured contrast data and the fitted values of ${\sigma }_{\phi }^{i}$ for all fully-entangling *XX* gates on the ion pairs used in this work.

In the noise-model simulations, for each shot, we sample random numbers *s* from the standard normal distribution ${{{\mathcal{N}}}}(0,1)$. When executing quantum circuits, we replace the ideal *XX*(*θ*) gate by

22 $${{{{\rm{e}}}}}^{-\frac{{{{\rm{i}}}}}{2}\left(\theta {\hat{X}}_{i}{\hat{X}}_{j}+{\phi }_{i}{\hat{Z}}_{i}+{\phi }_{j}{\hat{Z}}_{j}\right)},\quad \,{{{\rm{where}}}}\,\,\,{\phi }_{i}=s\frac{{\sigma }_{\phi }^{i}| \theta | }{\pi /2}$$

is a rescaled phase shift on ion *i*.

## Supplementary information

Supplementary Information

Transparent Peer Review File

## Data Availability

All data shown in figures are publicly accessible in the following repository (10.5281/zenodo.20752984). The raw data generated in this study have been deposited in Duke University’s Box cloud storage database. The raw data are available under restricted access subject to intellectual property laws, non-disclosure agreements, and trade-secret protections, in accordance with all applicable laws, regulations, agreement terms and conditions, and the policies and directives of the authors’ sponsors and affiliated institutions. Access can be obtained by contacting Marko Cetina.

## Code availability

The MERA code used in this work is publicly accessible in the following repository (10.5281/zenodo.20690576).
