# Supplementary material for: Probing entanglement scaling across a quantum phase transition on a quantum computer
Source: Nat Commun. 2026 Jul 27;17:9110. doi: 10.1038/s41467-026-75251-x (PMC13518892; doi:10.1038/s41467-026-75251-x)
Supplement: Supplementary file 1 [file 41467_2026_75251_MOESM1_ESM.pdf]

Supplementary Information:
Probing entanglement scaling across a quantum phase transition on a quantum computer

Qiang Miao,[1] Tianyi Wang,[1,2] Kenneth R. Brown,[1,2,3,4] Thomas Barthel,[1,2,5,*] and Marko Cetina[1,2,3,†]

[1]*Duke Quantum Center, Duke University, Durham, North Carolina 27701, USA*
[2]*Department of Physics, Duke University, Durham, North Carolina 27708, USA*
[3]*Department of Electrical and Computer Engineering, Duke University, Durham, North Carolina 27708, USA*
[4]*Department of Chemistry, Duke University, Durham, North Carolina 27708, USA*
[5]*National Quantum Laboratory, University of Maryland, College Park, MD 20742, USA*
(Dated: April 2026)

Supplementary Note 1. Gate Compilation and Circuit Examples

In the main text, we showed tensor network diagrams of MERA and schematic gate decompositions of individual tensors. Here, we demonstrate how to compile these into native quantum gates and provide concrete circuit examples for measuring both local observables and nonlocal entanglement properties.

As shown in Fig. S1a, a generic 4-by-4 unitary operation can be decomposed into a two-qubit circuit using 3 entangling gates and 12 single-qubit gates, involving a total of 15 parameters (up to a global phase). However, this generic decomposition turns out to be unnecessary for our MERA circuits. For example, in a network of such two-qubit unitaries, redundant single-qubit gates on connected bonds can be eliminated. More importantly, a physically motivated ansatz should preserve the symmetries of the Hamiltonian, except in cases of spontaneous symmetry breaking.

For the transverse-field Ising model studied here, the ground state exhibits time-reversal symmetry (invariance under complex conjugation in the computational basis) for all values of g and a $\mathbb{Z}_2$ symmetry when $g \leq 1$. While a global symmetry does not necessarily imply local symmetry, we find that, in our case, it does hold locally. A suitable choice of entangling gates that respect the symmetries of the Ising model includes $XY(\theta) = \mathrm{e}^{-\mathrm{i}\theta \hat{X}\otimes\hat{Y}/2}$ and $YX(\theta) = \mathrm{e}^{-\mathrm{i}\theta \hat{Y}\otimes\hat{X}/2}$,

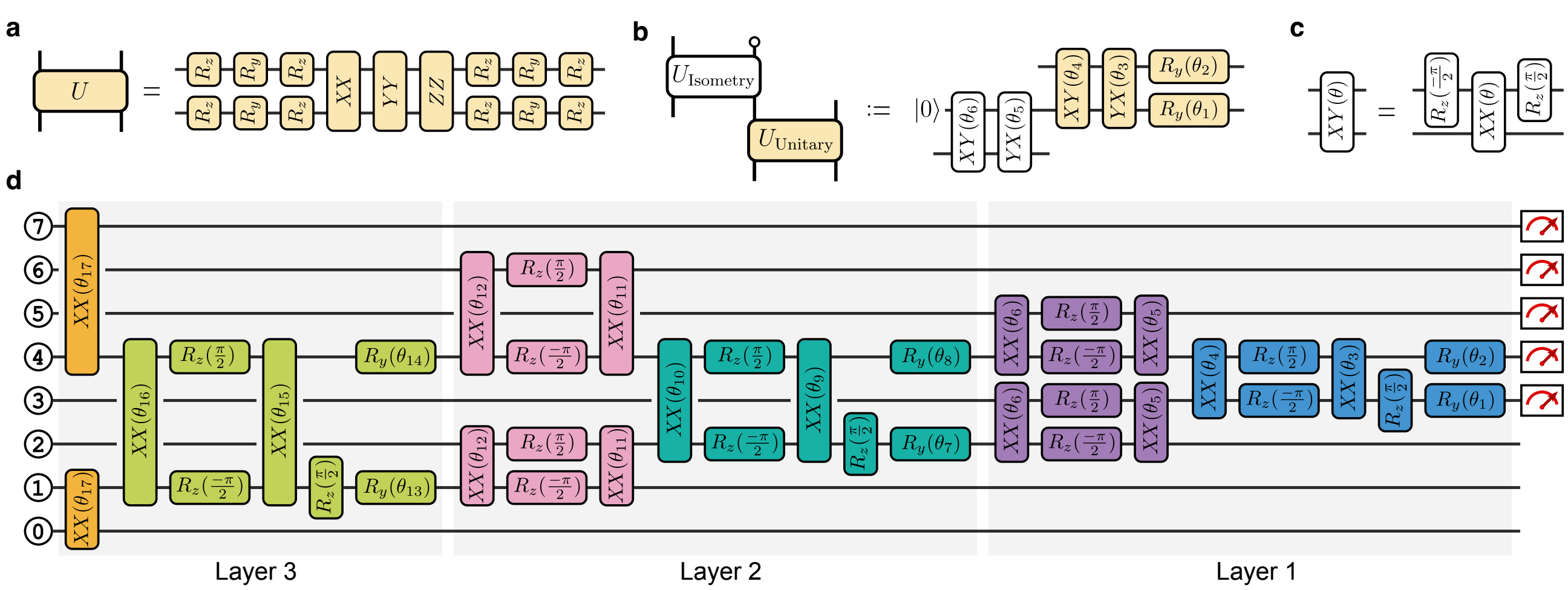

FIG. S1. **Gate compilation and circuit examples.** **a** Gate decomposition of a generic two-qubit unitary. **b** Gate compilation for a unit cell in the binary 1D MERA, incorporating symmetry constraints. The isometry tensor is formed by injecting a $|0\rangle$ qubit (indicated by a circle) into one leg of a unitary tensor. The unit cell contains six variable gates. **c** Equivalence between the XY gate and a circuit of three native gates. **d** An example circuit used in the experiment, composed of native gates. Qubits (circles with indices) are initialized in the $|0\rangle$ state. The circuit consists of three layers, and in each layer, gates of the same color correspond to either two isometric tensors or a single unitary (dis)entangler, respectively. Local observables at spin sites 0 and 1 are obtained by measuring qubits 3 and 4, while subsystem entanglement is extracted by measuring qubits 5, 6, and 7.

---

* thomas.barthel@duke.edu
† marko.cetina@duke.edu

which are real in the computational basis and respect the model's $\mathbb{Z}_2$ symmetry as $[\otimes_i \hat{Z}_i, XY(\theta)] = 0$. Based on this, we replace XX and YY gates with XY and YX gates. We remove ZZ and single-qubit Z gates as they violate time-reversal symmetry. Variable $R_y(\theta) = \mathrm{e}^{-\mathrm{i}\theta\hat{Y}/2}$ gates are retained in some tensors to allow for spontaneous breaking of the $\mathbb{Z}_2$ symmetry. The R_y gates that have been removed only have a relatively small effect on the accuracy.

The resulting refined gate compilation for a unit cell in the binary MERA, including one isometry and one unitary (dis)entangler, is shown in Fig. S1b. This symmetry-respecting compilation significantly reduces the parameter space and redundancy without sacrificing expressiveness in approximating the ground state, as confirmed by our numerical simulations. Since binary MERA with one qubit per bond is classically simulable, we do not implement the variational MERA on the quantum computer. Instead, we classically optimize the MERA with the gate configuration in Fig. S1b. In the classical optimization procedure, convergence is reached when the energy density no longer decreases and fluctuates only within a small tolerance (typically on the order of 10^{-8} or below). We find that the optimization process is robust and yields consistent results across different initial conditions. Examining these optimized parameters, we note that while the R_y gate is retained for all values of g, the optimized angles are non-zero only when $g < 1$ within our data points, which is consistent with the presence of a non-zero order parameter in the ferromagnetic phase.

In our experimental setup, the native entangling gate is the XX gate. As shown in Fig. S1c, we convert the XY and YX gates into native gates by appending additional virtual $R_z(\pm\frac{\pi}{2})$ rotations, which are effectively free, as mentioned in the main text.

As previously discussed and shown in Fig. 1, local observables and subsystem entanglement can be probed by preparing only the causal cone state (unshaded regions in Figs. 1 and 5), rather than the full MERA circuit. Figure S1d presents a concrete example of such a circuit. From left to right, 8 qubits are initialized in the $|0\rangle$ state, followed by layers of gates grouped into either isometry or (dis)entangler tensors. Finally, the desired observables are measured. In this circuit example, measurements on qubits 3 and 4 yield local observables on spin sites 0 and 1 as indicated in Fig. 1, while qubits 5, 6, and 7 are used to evaluate the entanglement between the subsystems A and B consisting of spin sites $(-\infty, 1]$ and $[2, \infty)$, respectively.

Supplementary Note 2. Variational optimization of MERA circuits

As discussed in the main text, since the 1D MERA circuits utilized in our experiment are classically simulable, we optimized them via classical computation. However, the classical numerical cost of tensor contractions increases sharply for dimensions $D > 1$. By replacing these classical computations with quantum circuits on a quantum processor, a hybrid optimization of MERA circuits offers a scalable pathway for the investigation of many-body ground states. To demonstrate the feasibility of this framework and explore its behavior under realistic experimental conditions, we present a corresponding numerical simulation of the hybrid variational optimization.

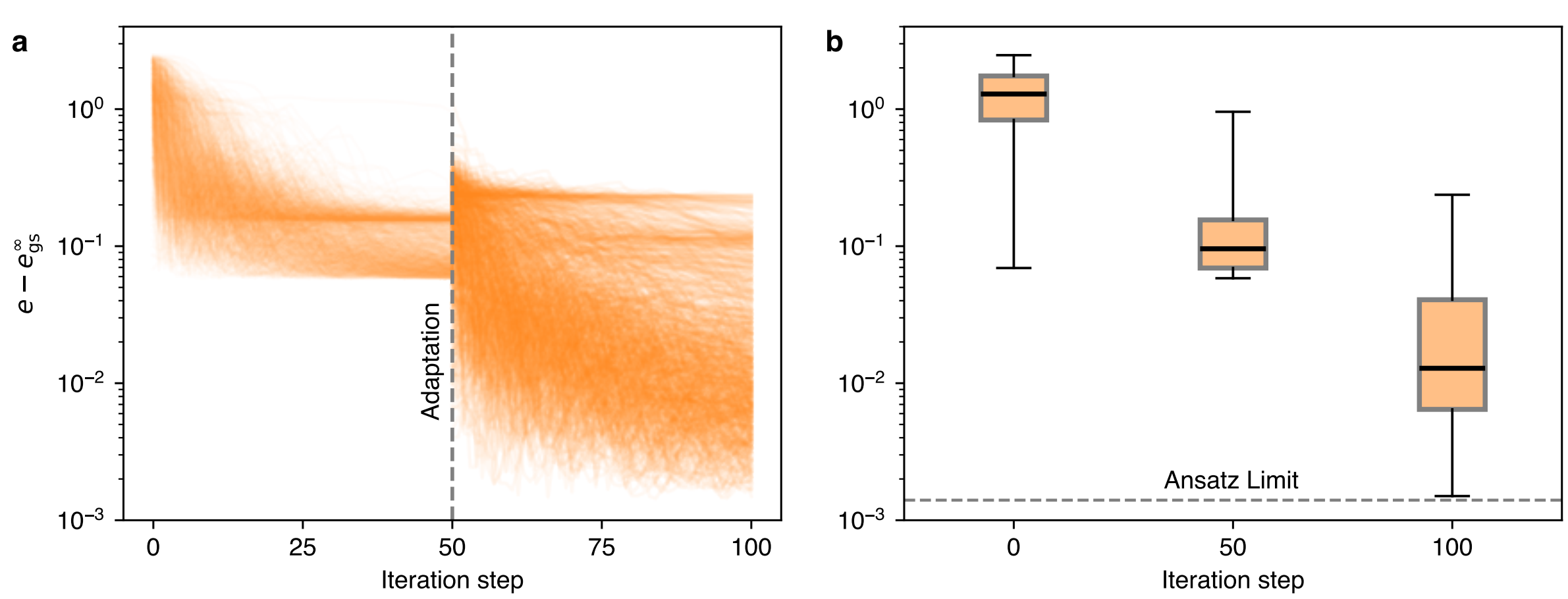

FIG. S2. **Simulation of hybrid variational optimization.** **a** Energy-density error $e - e_{\mathrm{gs}}^{\infty}$ depending on the iteration step for 1,000 randomly initialized MERA circuits. **b** Quantile distributions at different optimization steps. The whisker end caps indicate the minimum and maximum values, the box edges denote the 25% and 75% quantiles, and the black line marks the median. The gray dashed line indicates the ansatz limit after adaptation (second stage).

As a concrete example, we consider the critical point $g = 1.0$ of the 1D Ising model and apply a scale-invariant MERA

ansatz. The true ground state at criticality is strongly correlated and challenging to approximate. Nevertheless, a scale-invariant MERA with the smallest bond dimension $\chi = 2$ can already accurately represent this state, achieving an energy-density error as low as 1.4×10^{-3} in noiseless numerical simulations.

To reflect experimental conditions, our variational optimization simulations explicitly incorporate measurement uncertainty by sampling measurement outcomes rather than using exact energies. To reduce the sampling complexity, we employ the SPSA (Simultaneous Perturbation Stochastic Approximation) optimizer instead of standard gradient descent, which requires evaluating the energy at only two parameter points per iteration.

Figure S2a shows the energy-density error during optimization for 1,000 randomly initialized MERA circuits. To improve convergence and reduce resource costs, we adopt an adaptive strategy. During the first 50 iterations, we optimize a single-layer MERA ($T = 1$) using only 100 shots per measurement basis. Since the Ising Hamiltonian only involves the Z and X bases, each iteration requires 400 shots in total to evaluate the gradient and determine the move direction. In this stage, the energy error typically reaches the 0.1–0.2 range, consistent with the shot-noise limit. In the subsequent 50 iterations, we increase the number of MERA layers to $T = 6$ and the shot count to 10,000 per measurement basis. By the end of this stage, most trajectories converge to energy errors on the order of 10^{-2}, again consistent with shot-noise limit. Figure S2b shows the corresponding quantile distributions at different optimization steps. At the end of optimization, at least 25% of the trajectories achieve an error below 0.01 for the energy density and, in the best cases, the optimization reaches the intrinsic ansatz limit.

While it remains challenging to make quantitative predictions for the optimization cost of MERA circuits in 2D systems, these 1D results suggest that, with appropriate optimizer choices and warm-up strategies, the number of quantum states required for optimization is comparable to the shot count needed to reach a desired target accuracy.

Supplementary Note 3. Simulating XXZ chain by MERA circuits

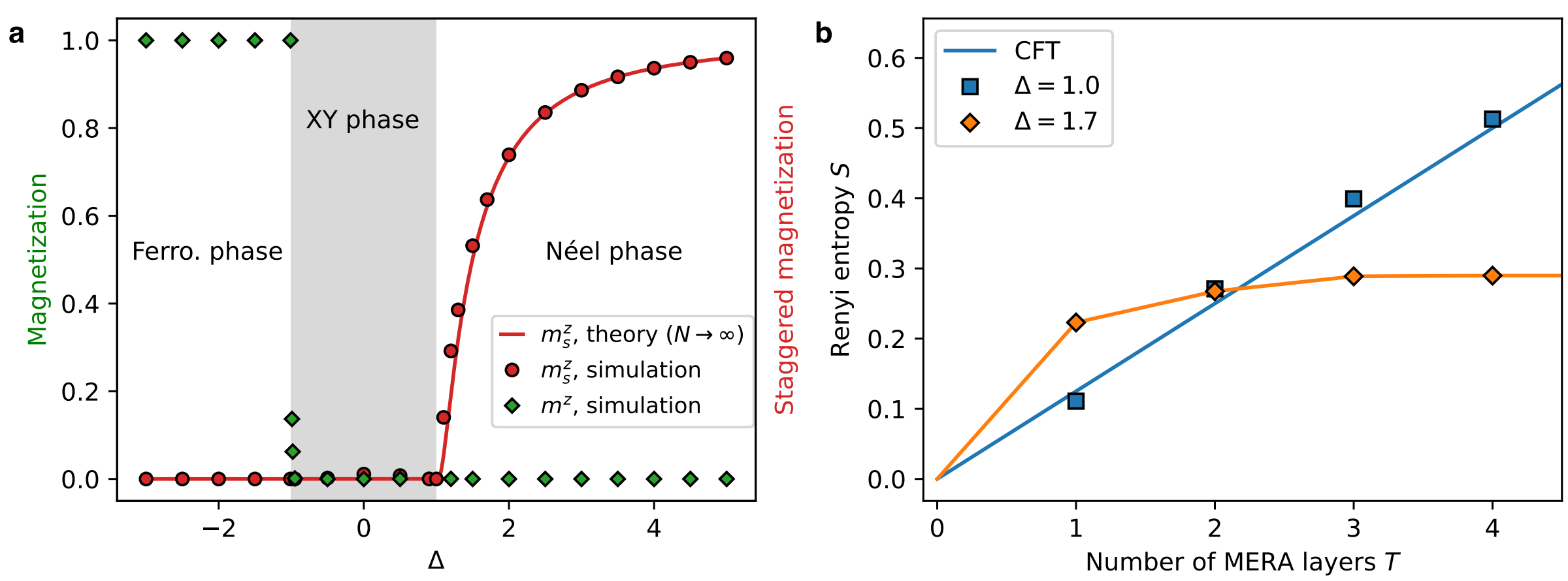

FIG. S3. **XXZ chain simulated via quantum MERA circuits.** (a) The staggered magnetization m_s^z serves as an order parameter for the $\mathbb{Z}_2$ symmetry breaking when $\Delta > 1$. The uniform magnetization m^z equals 1 in the ferromagnetic phase and 0 otherwise. (b) Entanglement scaling at and away from criticality. The solid line represents the conformal field theory (CFT) prediction across the entire XY phase, while the dots show the scaling obtained from MERA circuits.

While we primarily demonstrate our approach using the Ising model, the framework is broadly applicable. To illustrate this, we apply it to the 1D XXZ model

$$\hat{H} = \sum_i (\hat{S}_i^x \hat{S}_{i+1}^x + \hat{S}_i^y \hat{S}_{i+1}^y + \Delta \hat{S}_i^z \hat{S}_{i+1}^z). \tag{1}$$

The XXZ spin chain exhibits a quantum phase transition at the isotropic point $\Delta = 1.0$, separating a gapless critical XY (Luttinger-liquid) phase from a gapped Néel antiferromagnetic phase. This transition is of the Berezinskii-Kosterlitz-Thouless (BKT) type, and it is less conventional than the Ising case. At $\Delta = -1$ the system transitions to a (trivial) ferromagnetic phase.

Accurately describing more complex models requires larger MERA bond dimensions. We numerically simulate the XXZ model by designing MERA quantum circuits where we assign two qubits to each bond; this yields a bond dimension of $\chi = 4$, doubling the qubit resources compared to the Ising case. To implement the enlarged tensors efficiently, we

impose an internal substructure comprising brick-wall steps of two-qubit blocks, following our previous work [1]. Each generic two-qubit block is parameterized by the unitary operator $U = [R_z(\theta_1) \otimes R_z(\theta_2)]R_{zz}(\theta_3)R_{yy}(\theta_4)R_{xx}(\theta_5)$ [2]. Using MERA circuits with three steps per tensor and up to five layers, we obtain well-behaved order parameters and entanglement entropy scaling in the XXZ model, as shown in Fig. S3. In panel (a), the MERA circuit reproduces the sharp changes in order parameters near the phase transitions, analogous to Fig. 2a. More importantly, Fig. S3b can resolve logarithmic entanglement scaling at $\Delta = 1.0$ and area-law scaling at $\Delta = 1.7$. Although the resource requirements of this XXZ implementation (24 qubits and 540 native two-qubit gates) exceed the capabilities of our current experimental setup, they are accessible in other state-of-the-art quantum hardware. For example, circuits with more than 2,000 two-qubit gates and 56 qubits have been demonstrated on the Quantinuum H2 system (Ref. [3]).

Supplementary Note 4. State Preparation and Measurement Error

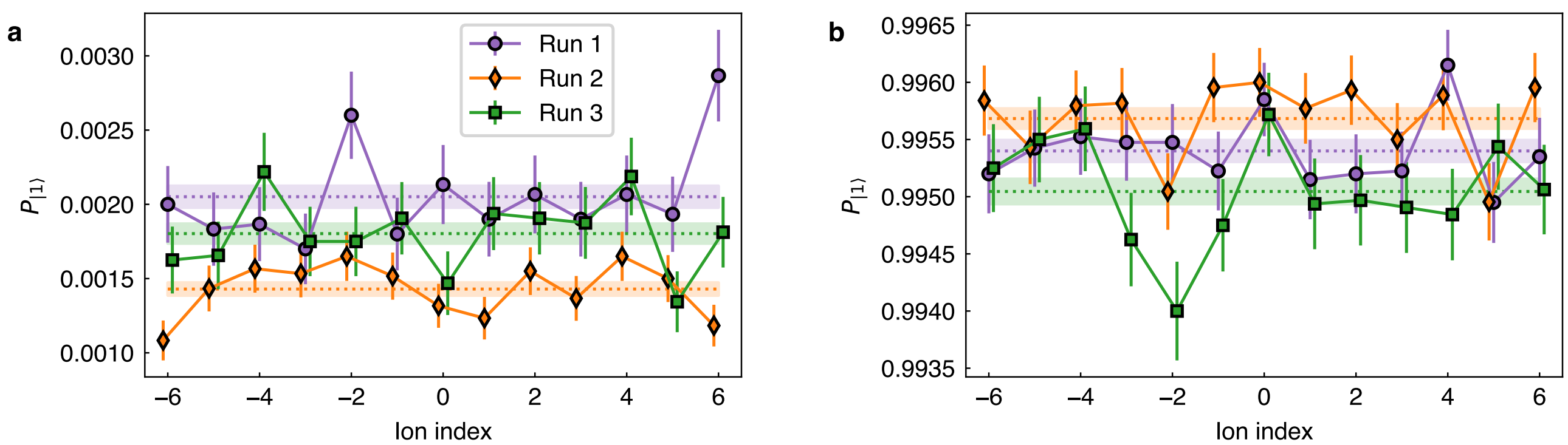

FIG. S4. **State preparation and measurement (SPAM) population as a function of the ion index. a** Dark-state ($|0\rangle$) SPAM experiments, with ion-averaged errors of 0.205(7)%, 0.143(4)%, and 0.180(7)% for three runs. **b** Bright-state ($|1\rangle$) SPAM experiments, with ion-averaged errors of 0.460(9)%, 0.432(9)%, and 0.495(11)% for three runs. Error bars and shaded areas indicate 1σ statistical intervals.

Figure S4 shows run-by-run results of state preparation and measurement (SPAM) on different experimental days. As mentioned in the Methods, preparing the bright state requires an additional $R_y(\pi)$ gate. We checked the rotation error of the $R_y(\pi)$ gates by applying nine consecutive $R_y(\pi)$-gates, yielding a population error of $0.56^{+0.17}_{-0.12}\%$ as determined by the binomial proportionate 95% confidence interval using the Clopper-Pearson method. The population error resulting from single-qubit gates should scale superlinearly with the number N of gates, corresponding to a mixture of uncorrelated and correlated noise. Our results imply a population error below 10^{-3} per π gate, which is well below the determined average bright-state SPAM error of 0.45%.

The measurement error for the $^{171}Yb^+$ hyperfine qubits arises from optical pumping between the hyperfine states during the optical detection pulse [4, 5]. We fit the measured photon count statistics at various detection time τ to an optical pumping model as discussed below to determine the state preparation error and the measurement errors.

The distributions of detection counts for a single ion prepared in the bright state for different detection times are shown in Fig. S5a. Ideally, the ion would cycle between the $|S, F = 1\rangle$ and $|P, F = 0\rangle$ manifolds, resulting in a Poisson distribution of the recorded photon counts. However, the bright state can be off-resonantly pumped to the dark hyperfine ground state via the $|S, F = 1\rangle \rightarrow |P, F = 1\rangle$ transition detuned by 2.1 GHz with a rate R_b that is about 5×10^4 times lower than the bright-state photon scattering rate $\Gamma_{\rm sc}$. Neglecting the even slower dark-to-bright pumping process, the predicted distribution of the bright-state photon counts are

$$P_b(n) = \mathrm{e}^{-R_b\tau}\mathcal{P}(n;\eta\Gamma_{\rm sc}\tau) + \int_0^\tau \mathrm{e}^{-R_bt}\mathcal{P}(n;\eta\Gamma_{\rm sc}t)\, R_b \;\mathrm{d}t, \tag{2}$$

where $\mathcal{P}(n;\bar{n}) = e^{-\bar{n}}\bar{n}^n/n!$ is the Poisson distribution with mean $\bar{n}$, and η is the detection efficiency. The red dashed curves in Fig. S5a show fits of the obtained count data to this model with $\eta\Gamma_{\rm sc}\tau$ and R_b as the fitting parameters. We average the fitted R_b values with weights inversely proportional to the fitted variances to obtain $R_b = 3.0(1) \times 10^2$/s. The detection count statistics for a single ion prepared in the dark $|S, F = 0\rangle$ state are shown in Fig. S5b. The dark state can be off-resonantly pumped to the bright manifold via the 14.7-GHz detuned $|S, F = 0\rangle \rightarrow |P, F = 1\rangle$ transition, with a rate R_d that is about 10^6 times lower than $\Gamma_{\rm sc}$. Neglecting the reverse pumping process yields the

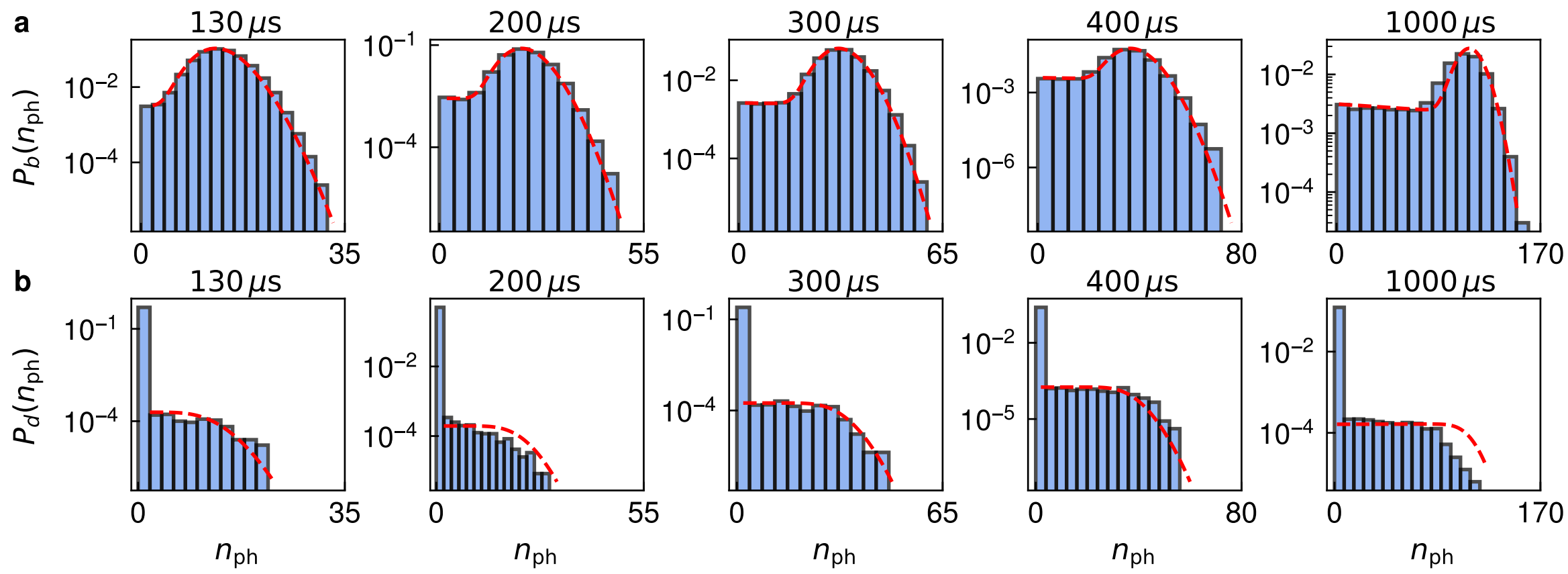

FIG. S5. Distribution of the detected photon counts $n_{\rm ph}$ for different detection times for the bright state (**a**) and the dark state (**b**). The red dashed lines indicate a fit to the models from Eq. (2) and (3).

predicted dark-state count distribution

$$P_d(n) = \int_0^\tau \mathcal{P}(n; \eta\Gamma_{\rm sc}(\tau - t))\, e^{-R_d t} R_d \, \mathrm{d}t. \tag{3}$$

Fitting this model to the count data for photon number $n_{\rm ph} > 1$ in Fig. S5b yields $R_d = 18(1)$/s. The obtained ratio of R_b and R_d is close to the theoretical prediction in Ref. [6].

Supplementary Note 5. Idle Dephasing

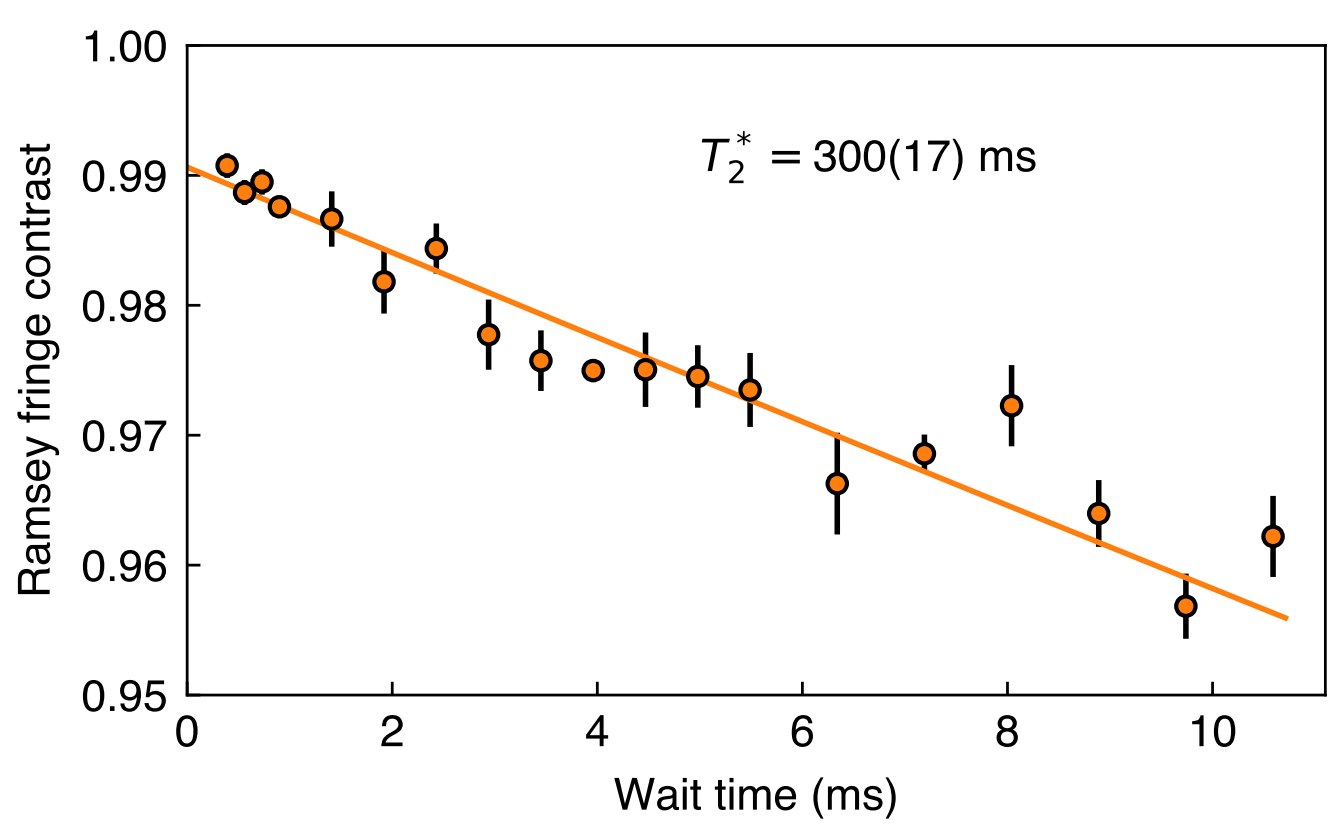

FIG. S6. The average qubit Ramsey fringe contrast as a function of the idle time $\tau_{\rm idle}$ from a sample dataset. The orange line represents the fit to the model $A\,\mathrm{e}^{-\tau_{\rm idle}/T_2^*}$ yielding $T_2^* = 300(17)$ ms and $A = 0.991(1)$. For the experimental data, error bars indicate 1σ fitting uncertainties. The deviation of the fitted value of A from 1 mainly reflects SPAM errors, with additional contributions potentially arising from residual single-qubit gate errors and qubit addressing and detection crosstalk.

Supplementary Note 6. Breakdown of error contributions

To better understand our experimental results, we analyze the breakdown of error contributions through numerical simulation. As shown in Fig. S7, we selected two representative cases from Fig. 4 of the main text, one with the highest reported infidelity $1 - \mathcal{F}$ ($\sim 10^{-2}$) and one with the lowest ($\sim 10^{-4}$).

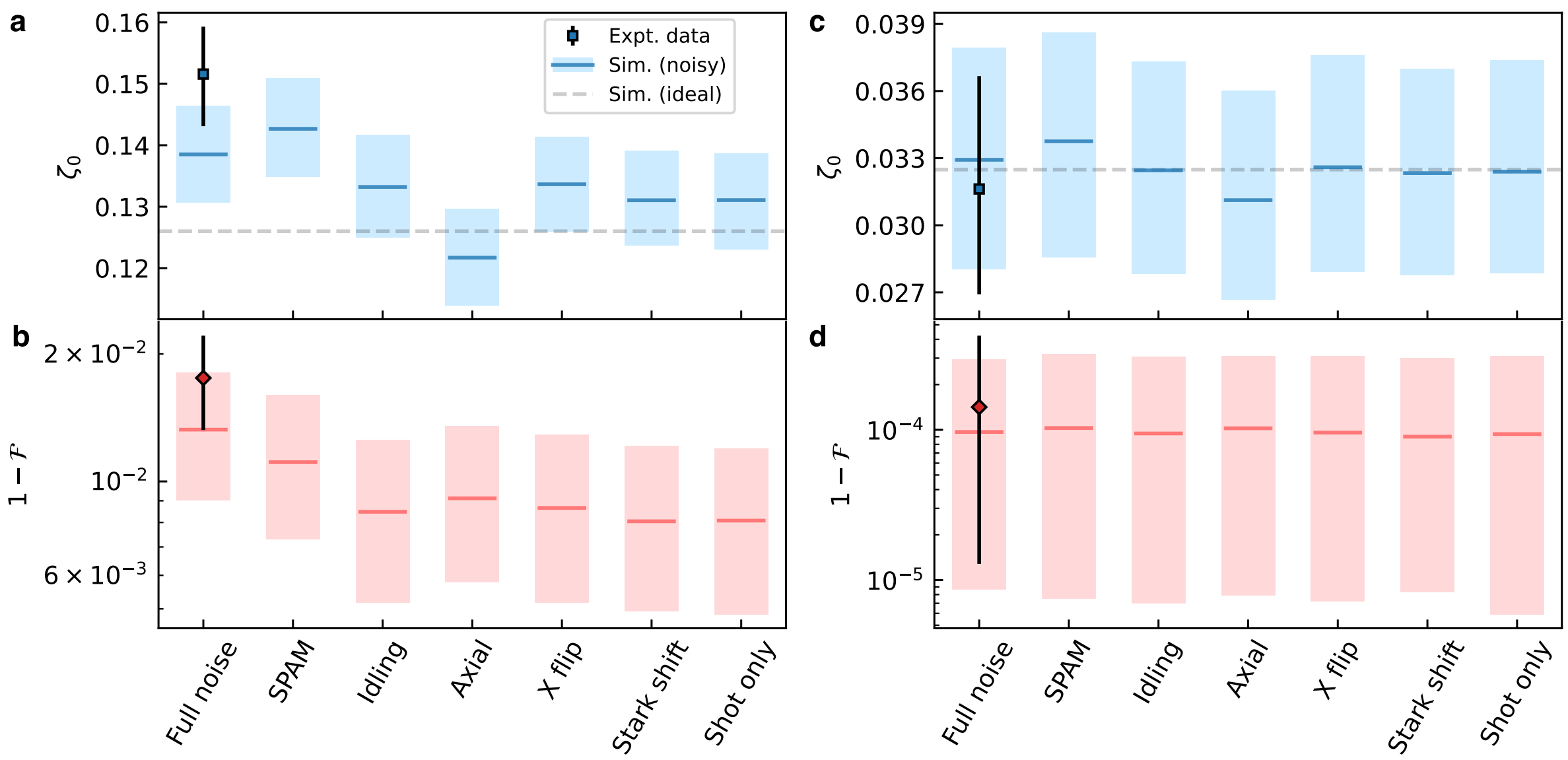

FIG. S7. **Breakdown of error contributions.** The top row (**a**, **c**) shows the first eigenvalues ζ_0 of the entanglement Hamiltonian, and the bottom row (**b**, **d**) shows corresponding state infidelities $1-\mathcal{F}$. The left column (**a**, **b**) presents results at the critical point $g=1$ with $T=4$, while the right column (**c**, **d**) corresponds to the gapped phase at $g=1.5$ with $T=1$. Experimental data points with error bars are reproduced from Fig. 4 in the main text. The dashed lines indicate the ideal simulation results from Fig. 4. Noisy simulation results are shown as shaded regions representing the 95% quantiles, with solid colored lines indicating the mean values. In the "full noise" case, we reproduce the full noisy simulation including all error sources from Fig. 4. In the "shot only" case, all errors are disabled except for shot noise (the statistical uncertainty due to the finite number of measurement shots). In the remaining cases, the simulations include both the shot noise and the specified individual error source. We run 1000 independent noisy simulation trials for each case. Each trail uses the same number of measurement shots as the experiment: 1,500 shots per basis per trial in the left column, and 8,000 shots per basis per trial in the right column.

The case of highest reported infidelity corresponds to a 4-qubit tomography result on a 4-layer MERA circuit at the critical point $g=1$. The circuit involves 9 qubits and 21 XX gates with rotation amplitudes ranging from 0.06π to 0.18π. We performed 1,500 measurement shots per basis over a total of $3^4=81$ measurement bases. Based on our error model and the calibrated parameters provided in Methods, we identify several key sources of error. First, there is the inevitable statistical uncertainty in the measured density matrix elements on the order of $\sim 10^{-2}$ from the finite number of measurement shots. Second, aggregated SPAM errors for the 4-qubit tomography are on the order of $\sim 10^{-2}$. The dominant gate error arises from rotation-angle fluctuations caused by axial motion of the ion chain, which, for fully-entangling gates, typically leads to errors on the order of 10^{-2}. After accounting for the error rescaling due to smaller rotation angles in the actual MERA gates and the accumulation of errors throughout the circuit, the overall gate-induced error is also expected to be in the 10^{-2} range. This estimate is confirmed in Fig. S7a. A small discrepancy remains between the experimental data and the full-noise simulation, which we attribute to system miscalibration over the long experimental runtime required for 4-qubit tomography, as well as unmodeled single-qubit gate errors.

The case of lowest reported infidelity corresponds to a single-qubit tomography result on a single-layer MERA circuit in the gapped phase at $g=1.5$. The circuit involves only 2 qubits and a single XX gate with a rotation amplitude of 0.095π. We performed 8,000 measurement shots for each Pauli-X, -Y, and -Z basis. In this case, the statistical uncertainty from finite sampling is on the order of $10^{-3}\sim10^{-2}$, while the SPAM error for a single qubit is on the order of 10^{-3}, and the error from the single gate is also on the order of 10^{-3}. Hence, we expect the statistical uncertainty to dominate over other error sources, which is consistent with the simulation results shown in Fig. S7c.

We observe that the infidelity is of comparable magnitude to the errors for the high-infidelity case in Fig. S7b and at least an order of magnitude below the error scale for the low-infidelity case in Fig. S7d. We attribute this to the fact that the infidelity has different scaling behavior for rank-deficient and full-rank states: Consider the exact (noise-free) density operator $\hat{\rho}$ and the perturbed version $\hat{\sigma}=\hat{\rho}+\epsilon\hat{D}$ with noise level ϵ and $\mathrm{Tr}(\hat{D})=0$. For a full-rank state $\hat{\rho}$, the perturbed $\hat{\sigma}$ remains well-defined (positive semidefinite) for sufficiently small $\epsilon>0$ as well as small $\epsilon<0$. The infidelity $1-\mathcal{F}(\hat{\rho},\hat{\sigma}(\epsilon))$ is differentiable and minimal at $\epsilon=0$. This implies that the linear term vanishes and

$1-\mathcal{F}=\mathcal{O}(\epsilon^2)$. This is the case in Fig. S7d, where the corresponding single-qubit reduced density matrix has full rank with eigenvalues $\{0.98, 2.2\times10^{-2}\}$. A linear scaling $1-\mathcal{F}=\mathcal{O}(\epsilon)$ is only possible for rank-deficient states. For example, consider a pure state $\hat{\rho}=|\psi\rangle\langle\psi|$ and the perturbation $\hat{D}=-|\psi\rangle\langle\psi|+|\psi_\perp\rangle\langle\psi_\perp|$ with an orthogonal state $|\psi_\perp\rangle$ and $\epsilon\geq 0$. In this case, the infidelity is simply ϵ. In the current experiments, the ideal reduced density matrix is indeed approximately rank-deficient at larger layer numbers T, and this is the case in Fig. S7b with eigenvalues $\{0.92, 7.9\times10^{-2}, 3.5\times10^{-3}, 3.1\times10^{-4}, 2.7\times10^{-4}, 2.7\times10^{-5},\dots\}$.

[1] Q. Miao and T. Barthel, Quantum-classical eigensolver using multiscale entanglement renormalization, Phys. Rev. Res. **5**, 033141 (2023).

[2] To maximize compatibility with our specific gate parameterization, we apply a local basis transformation (alternating $\hat{X}$ flips on the lattice) that maps the standard XXZ Hamiltonian to $\hat{H}=\sum_i(\hat{S}^x_i\hat{S}^x_{i+1}-\hat{S}^y_i\hat{S}^y_{i+1}-\Delta\hat{S}^z_i\hat{S}^z_{i+1})$.

[3] R. Haghshenas, E. Chertkov, M. Mills, W. Kadow, S.-H. Lin, Y. H. Chen, C. Cade, I. Niesen, T. Begušić, M. S. Rudolph, C. Cirstoiu, K. Hémery, C. Mc Keever, M. Lubasch, E. Granet, C. H. Baldwin, J. P. Bartolotta, M. Bohn, J. J. Burau, J. Cline, M. DeCross, J. M. Dreiling, C. Foltz, D. Francois, J. P. Gaebler, C. N. Gilbreth, J. Gray, D. Gresh, A. Hall, A. Hankin, A. Hansen, N. Hewitt, C. A. Holliman, R. B. Hutson, M. Iqbal, N. Kotibhaskar, E. Lehman, D. Lucchetti, I. S. Madjarov, K. Mayer, A. R. Milne, S. A. Moses, B. Neyenhuis, G. Park, A. R. Perry, B. Ponsioen, M. Schecter, P. E. Siegfried, D. T. Stephen, B. G. Tiemann, M. D. Urmey, J. Walker, A. C. Potter, D. Hayes, G. K.-L. Chan, F. Pollmann, M. Knap, H. Dreyer, and M. Foss-Feig, Digital quantum magnetism on a trapped-ion quantum computer, Nature **653**, 56 (2026).

[4] S. Crain, C. Cahall, G. Vrijsen, E. E. Wollman, M. D. Shaw, V. B. Verma, S. W. Nam, and J. Kim, High-speed low-crosstalk detection of a 171yb+ qubit using superconducting nanowire single photon detectors, Commun Phys **2**, 1 (2019).

[5] C. Roman, A. Ransford, M. Ip, and W. C. Campbell, Coherent control for qubit state readout, New J. Phys. **22**, 073038 (2020).

[6] R. Noek, G. Vrijsen, D. Gaultney, E. Mount, T. Kim, P. Maunz, and J. Kim, High speed, high fidelity detection of an atomic hyperfine qubit, Opt. Lett., OL **38**, 4735 (2013).
