# Supplementary material for: Probing entanglement scaling across a quantum phase transition on a quantum computer
Source: Nat Commun. 2026 Jul 27;17:9110. doi: 10.1038/s41467-026-75251-x (PMC13518892; doi:10.1038/s41467-026-75251-x)
Supplement: Supplementary file 2 [file 41467_2026_75251_MOESM2_ESM.pdf]

nature portfolio

Peer Review File

Probing entanglement scaling across a quantum phase transition on a quantum computer

Corresponding Author: Professor Marko Cetina

This file contains all reviewer reports in order by version, followed by all author rebuttals in order by version.

Version 0:

Reviewer comments:

Reviewer #1

(Remarks to the Author)
Dear editor,

the manuscript "Probing entanglement scaling across a quantum phase transition on a quantum computer" discusses the implementation of a tensor network ansatz (MERA) strategy on an ion-based digital quantum computer to probe entanglement properties and quantum correlations of the 1D Ising model via hybrid classical-quantum methods.

I find the work well executed with high quality data and an in-depth precise analysis of the different aspects of the problem. However, I fail to see a sufficiently strong or groundbreaking aspect of the results or compelling evidence for publication in Nat.Communications. This is case especially because:

1) a demonstration of a similar strategy on an ion-based quantum computer has been already published [Haghshenas PRL 133, 266502, (2024)], although in that work only quantum correlations are computed whereas here a broader analysis is presented and also the interesting validation of the CFT log-area law by Calabrese&Cardy at criticality;

2) Ising model in 1D is a standard well established model, the properties of which can be computed with high accuracy using a variety of methods (including classical methods and experimental quantum computing platforms). A demonstration of the power of the method presented here in a different and more complex context for classical algorithms (e.g. a 2D system, out-of-equilibrium dynamics, less conventional critical points) would have been a very different story, proving the viability and practical application of the approach beyond the simplest toy model;

3) universal quantum computers are expected to overcome the computational overheads of classical methods. The employment of quantum-assisted classical approaches can raise some doubts, especially now that more coherent and large scale quantum devices are becoming available, which can target truly quantum algorithms. It is possible, however, that in certain conditions they can be used to boost classical computations, but a concrete demonstration (see 2) is needed.

For the reasons above, mostly 1) and 2), I find this work as a technically well-executed analysis of a quantum-assisted classical algorithm for the study of critical properties of the conventional Ising model, based on established methodologies and extending previously demonstrated (or proposed) analogous approaches on the same model. The work is mostly of value for those studying/testing these approaches, and thus I see it more appropriate to publish it in a specialized journal.

I have a few remarks on the content:

- What are the main reasons why MPS-based approaches are not applicable or useful here? I believe mentioning MPS ansatzes and discussing a comparison with them is necessary here, even if this has been done already in the literature. What features of MPS do not allow to implement a scheme similar to the one used in this work? The absence of causal structure? a more limited compression? the scaling of certain quantities? A paragraph explaining why a MERA-based computation for this problem is advantageous over MPS (if that is true) is necessary.

- In Sec. IIIA, the authors show properties in the symmetry broken phase with nonzero magnetization <X>. However, in a

symmetry broken phase there are two degenerate ground states. It is not clear how one of the two ferromagnetic ground states is attained instead of a generic superposition. A comment should be given.

- In Fig. 2b, the inset shows data indicating a critical exponent 1/8. In particular, the authors state in the text "The fitted values gradually converge to the theoretical prediction of 1/8", but I cannot see this well represented by the data shown, I actually do not see any convergence or actual quantitative correspondence with the critical value expected. I find it hard to conclude anything from the data shown.

Reviewer #2

(Remarks to the Author)
Miao and coworkers explore the ground-state properties of a 1d spin chain near a critical point using the multiscale entanglement renormalization ansatz to capture long-range correlations in the system. Towards this end, they perform experiments with a digital quantum simulator based on trapped ions for an investigation of a transverse-field Ising chain with a number of particles that is substantially bigger than the number of qubits of the simulator. They succeed in measuring not only local properties such as order parameter scaling, for which they find good agreement with known results, but also bipartite entanglement entropies and entanglement spectra by what they term holographic subsystem tomography. This approach works as the MERA ansatz enables an efficient representation of these quantities. Miao et al numerically optimized MERA circuits for representing ground states of the Ising model, and experimentally represented MERA states by subjecting the qubits to a sequence of entangling gates obtained from the numerical optimization. Information about the physical properties of the spin chain could then be extracted by measuring subsystems of the created quantum state.

To my knowledge, this is the first time that this approach has been implemented experimentally. The results look promising to me. They should be of interest to a wide range of researchers working in this field and can in principle be implemented on any experimental platform for digital quantum simulation.

For the model system investigated, exact results are known, which enables a comparison of the experimental findings with the ground truth. While computer simulations based on MERA circuits/states can be efficiently implemented for 1d systems, the authors stress that MERA representations of two-dimensional models come with high numerical costs of tensor contractions. The hope motivating the present experiments is that improved versions of the latter might have an edge over computer simulations of 2d models by swapping tensor contractions against quantum measurements. Whether this approach will work depends on the difficulty of variationally optimizing the quantum circuits that encode the MERA states. This is a question that the current manuscript does not answer. For this reason, I would have liked to learn about how difficult it would have been to find the MERA states representing ground-states of the 1d Ising model experimentally. While I would find it unreasonable to ask the authors to have their experiment answer this question, I feel that it would really strengthen this manuscript if the variational optimization of such a circuit was simulated numerically. How many quantum states would one have to generate in order to find the optimized gate sequence used in the experiments? Can one estimate how this number would scale when moving to a 2d system with a size where one could hope of experiments to predict system properties more precisely than numerical simulations?

Apart from this, I have hardly any comments:

* I would have like to find a bit more information about the extraction of critical exponents in Fig. 2b. Was a fit of the log of the mean magnetization vs the log of 1-g^2 used?

* Fig. 1d shows two different ion-to-qubit mappings but does not explain why they are expected to be optimal for the measurement of a particular quantity. Also, I couldn't figure out why in 1a and d some of the qubit numbers are printed in gray and others in black.

Overall, the results are convincing and significant. With the above clarifications and a brief trainability analysis, I would strongly support publication.

Reviewer #3

(Remarks to the Author)
This manuscript demonstrates the first observation of log-law scaling entanglement entropy at the quantum critical point of the Ising model, achieved through the implementation of MERA and holographic tomography. The results are supported by a thoughtful error model that closely aligns noisy simulations with experimental data, while the measured critical scaling and entanglement transition consistently reflect Ising universality predictions. The work is significant for overcoming fundamental finite-size limitations in quantum simulation and establishing a scalable framework for studying strongly-correlated systems. The analytical approach effectively exploits MERA's causal cone structure and scale-invariance properties to enable efficient characterization of critical phenomena with logarithmic resources, presenting a coherent and powerful framework for quantum many-body simulation. Methodologically, it leverages a fully-connected trapped-ion architecture with optimized qubit mappings and advanced gate control. However, additional details on experimental calibration and optimization procedures would further strengthen validity and clarity. I believe this manuscript is of broad interest and qualified to be published in nature communications. To increase the readability, I hope the authors could address the points below.

1. In Fig. 1a, do the circled numbers indicate the execution sequence of the quantum circuit used to prepare the MERA state? If so, why does this execution order not follow a layer-by-layer construction? In Fig 1.d, do the circled numbers have

the same meaning as those in Fig. 1a? What does the background color of each circle signify? A more detailed explanation of these annotations would greatly assist readers unfamiliar with the MERA formalism in deciphering these complex diagrams.

2. More detailed descriptions of the experimental implementation would improve the clarity of the research. Specifically:
(1) How is the strength of the transverse field g accurately calibrated and controlled in the setup?
(2) How are the parameters of the disentangler and isometry optimized, and how is the execution order of the quantum circuit determined? What key factors—such as noise resilience, resource constraints, or convergence criteria—are considered during the optimization process?
(3) How are ion reset and reinitialization procedures utilized across successive experimental runs to ensure comprehensive coverage of distinct causal cones? Furthermore, what is the cumulative impact of such reset-reinitialization cycles on the overall experimental error budget?

3. The authors observe that the small bond dimension χ leads to saturation of the order parameter near criticality (Fig. 2b). Besides, the finite correlation length resulting from the limited number of layers T may also contribute to the observed deviations. The manuscript would be strengthened by a more explicit discussion of both the robustness of qualitative conclusions, such as the logarithmic scaling of entropy, under these limitations, and the fundamental constraints on quantitative accuracy imposed by finite χ and T.

Version 1:

Reviewer comments:

Reviewer #1

(Remarks to the Author)
The authors have considered my criticism and pointed out how their results provides a significant advance in what has been done in the literature, also stressed by the opinions of the other referees. In particular they have stressed that one of the key highlights is the identification and characterization of entanglement scaling in a quantum device, a quantity that in previous works was out of reach. The authors have also shown how these methods apply to a distinct model, the XXZ model, where a gapless-to-gapped phase transition takes place. While this is only tested numerically, it provides a clear direction for applying this technique in generic many-body settings. The possibility to apply the method to higher-dimensional models is appealing, while not explored yet and its viability remains an open question at present. Besides scaling arguments, a concrete demonstration is still needed. Besides this, I am now more convinced that the manuscript is suited to publication in Nature Communications.

Reviewer #3

(Remarks to the Author)
I am satisfied with the authors reply to my questions. The results presented in this manuscript are interesting and scientifically significant. Therefore, I fully support the publication of this manuscript.

Open Access This Peer Review File is licensed under a Creative Commons Attribution 4.0 International License, which permits use, sharing, adaptation, distribution and reproduction in any medium or format, as long as you give appropriate credit to the original author(s) and the source, provide a link to the Creative Commons license, and indicate if changes were made.
In cases where reviewers are anonymous, credit should be given to 'Anonymous Referee' and the source.
The images or other third party material in this Peer Review File are included in the article's Creative Commons license, unless indicated otherwise in a credit line to the material. If material is not included in the article's Creative Commons license and your intended use is not permitted by statutory regulation or exceeds the permitted use, you will need to obtain permission directly from the copyright holder.
To view a copy of this license, visit https://creativecommons.org/licenses/by/4.0/

II Point-by-point Responses

Response to Report of Referee 1

Referee 1: I find the work well executed with high quality data and an in-depth precise analysis of the different aspects of the problem. However, I fail to see a sufficiently strong or groundbreaking aspect of the results or compelling evidence for publication in Nat. Communications.

Response: We thank the referee for the careful evaluation and the positive assessment of the technical quality of our work. We appreciate the concern about the scope and significance of the results. Please see our point-by-point response below.

Referee 1: 1) a demonstration of a similar strategy on an ion-based quantum computer has been already published [Haghshenas PRL 133, 266502, (2024)], although in that work only quantum correlations are computed whereas here a broader analysis is presented and also the interesting validation of the CFT log-area law by Calabrese & Cardy at criticality;

Response: We thank the referee for pointing out Ref. 28 [*PRL* **133**, 266502 (2024)] and for the opportunity to compare that work and ours. As correctly noted by the referee, Ref. 28 only measures two-local correlation functions at a single parameter point, and such observables are relatively straightforward to access experimentally.

In contrast, our work shows how the MERA approach enables small quantum devices to clearly resolve (i) a sharp many-body quantum phase transition (Figs. 2a and 2b) in terms of the order parameter and (ii) the associated different groundstate entanglement scaling laws (Figs. 3 and 4).

Experimentally resolving differences in ground-state entanglement scaling laws is highly challenging, as it requires access to a broad range of subsystem sizes ℓ, high-fidelity state preparation, and, without advanced tomography techniques, an exponentially large measurement cost ($\mathcal{O}(e^{\ell})$).

For example, the seminal work Ref. 17 [*Nature* **624**, 539 (2023)] could not distinguish the different scaling of the groundstate entanglement entropy at criticality ($\Delta = 1.0$) and away from criticality ($\Delta = 1.7$) (S_A^{VN} (blue) in Fig. 1c). As acknowledged in their main text: *"the small logarithmic correction to the entropy expected for a central charge $c = 1$ CFT is not conclusively observed for the low-energy state at $\Delta = 1$; experimental imperfections and noise presumably mask the associated weak long-range correlations."* This underlines the significance of our result: to the best of our knowledge, we are the first to experimentally distinguish distinct entanglement scaling laws across a quantum phase transition on a quantum device. The developed holographic tomography method will be a valuable tool for future research.

Referee 1: 2) Ising model in 1D is a standard well established model, the properties of which can be computed with high accuracy using a variety of methods (including classical methods and experimental quantum computing platforms). A demonstration of the power of the method presented here in a different and more complex context for classical algorithms (e.g. a 2D system, out-of-equilibrium dynamics, less conventional critical points) would have been a very different story, proving the viability and practical application of the approach beyond the simplest toy model;

Response: We agree with the referee that the 1D Ising model is a well-established model. We therefore chose this model to establish a proof-of-principle implementation, in a setting where theoretical requirements and experimental constraints can be carefully balanced and the correctness of the approach can be directly validated against known results.

We also agree that demonstrating the method in more complex contexts is an important and promising direction. Following the referee's suggestion, we have added data for the 1D XXZ model (Supplementary Note 4). The XXZ spin chain exhibits a quantum phase transition at the isotropic point ($\Delta = 1.0$), separating a gapless critical XY (Luttinger-liquid) phase from a gapped Néel antiferromagnetic phase. This transition is of the Berezinskii-Kosterlitz-Thouless (BKT) type, and it is less conventional than the Ising case.

Accurately describing more complex models requires larger MERA bond dimensions. We numerically simulate the XXZ model by designing MERA quantum circuits with two qubits per bond (bond dimension $\chi = 4$), doubling the qubit resources compared to the Ising case. Using MERA circuits involving up to 24 qubits and approximately 540 native two-qubit gates, we obtain accurate results for order parameters and

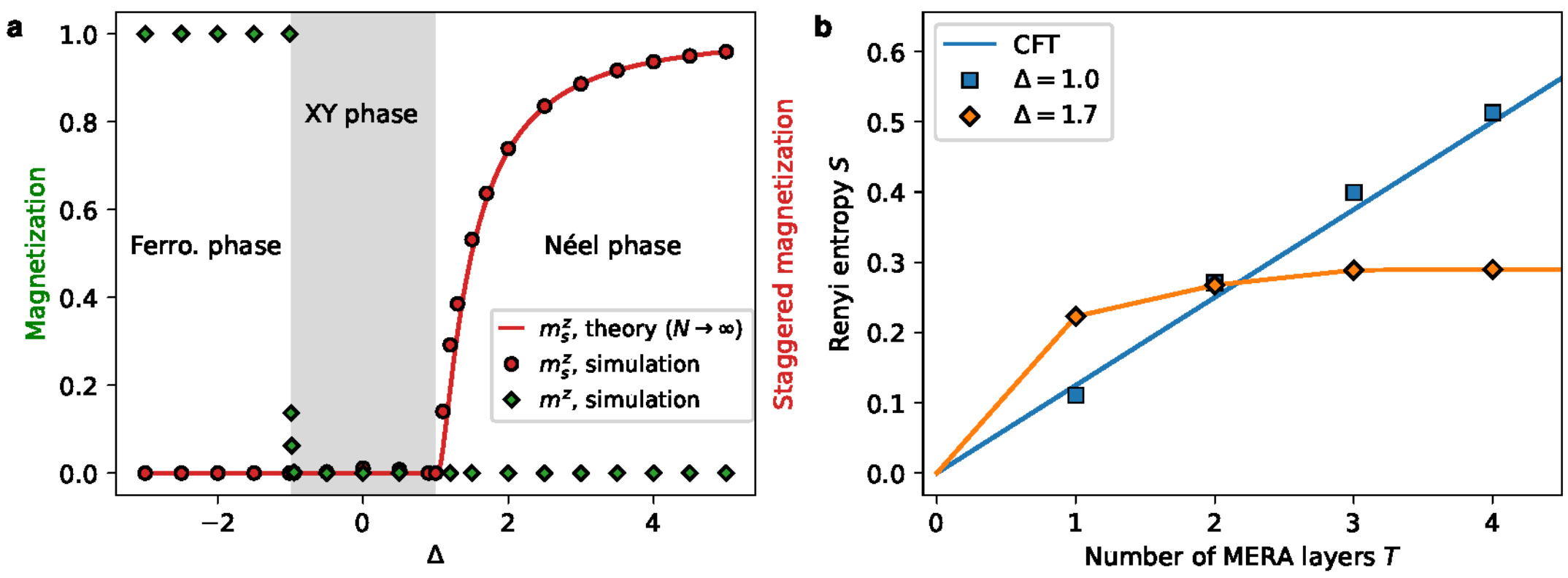

Figure R1: **XXZ chain simulated via quantum MERA circuits.** (a) The staggered magnetization m_s^z serves as an order parameter for the $\mathbb{Z}_2$ symmetry breaking when $\Delta > 1$. The uniform magnetization m^z equals 1 in the ferromagnetic phase and 0 otherwise. (b) Entanglement scaling at and away from criticality. The solid line represents the conformal field theory (CFT) prediction across the entire XY phase, while the dots show the scaling obtained from MERA circuits. [Reproduced from Fig. S5 in the updated manuscript (Supplementary Note 4).]

entanglement entropy in the XXZ model as shown in Fig. R1. In panel (a), the MERA circuit reproduces the sharp change in order parameters near the two phase transitions at $\Delta = -1$ and $\Delta = 1$, analogous to Fig. 2a. Entanglement data shown in panel (b) clearly resolves a logarithmic scaling at $\Delta = 1.0$ and area-law scaling at $\Delta = 1.7$. We specifically chose these values of Δ to match those studied in Ref. 17 [*Nature* **624**, 539 (2023)], where the distinct scaling behaviors were not experimentally resolved. Although the resource requirements of this XXZ implementation (24 qubits and 540 native two-qubit gates) exceed the capabilities of our current experimental setup, they are accessible in other state-of-the-art quantum hardware and will be available in our upgraded devices. For example, circuits with more than 2,000 two-qubit gates and 56 qubits have been demonstrated on the Quantinuum H2 system (Ref. 63).

We agree that out-of-equilibrium dynamics and extensions to two-dimensional systems are highly promising. These directions are the subject of ongoing upgrades in our experimental platform, but they remain beyond the scope of this work and take time to become feasible in our lab.

Referee 1: 3) universal quantum computers are expected to overcome the computational overheads of classical methods. The employment of quantum-assisted classical approaches can raise some doubts, especially now that more coherent and large scale quantum devices are becoming available, which can target truly quantum algorithms. It is possible, however, that in certain conditions they can be used to boost classical computations, but a concrete demonstration (see 2) is needed.

Response: We sincerely thank the referee for raising this important point regarding the trajectory of quantum computing and the role of hybrid algorithms. It is generally expected that most algorithms will be hybrid in nature requiring both classical and quantum computation. This is explicit in ideas for the design of catalysts with quantum computers [e.g., Phys. Rev. Research **3**, 03305 (2021)] but is even true of Shor's algorithm, where the output k/r of the quantum computer has to be classically processed to determine r. In a common formulation of Shor's algorithm with n digits, both the quantum period finding and the classical continued fraction expansion have time complexity $O(n^3)$.

Similarly, our hybrid quantum-classical MERA approach is not merely a stopgap measure, but a highly targeted method that directly addresses specific bottlenecks of classical and quantum computers, exploiting known properties of low-energy states in typical quantum many-body systems.

While more coherent, larger-scale devices are indeed becoming available, we are still operating within an era where noise and decoherence strictly limit circuit depth and direct mappings of many-body systems to the devices are hampered by strong finite-size effects. Fully coherent "truly quantum" algorithms for

groundstate preparation (such as quantum phase estimation, which comes with its own challenges) require deep circuits that remain out of reach for current hardware without full fault tolerance.

By contrast, our hybrid MERA approach leverages noise resilience, the general structure of groundstate correlations/entanglement in local many-body systems, and a clever embedding/mapping of large many-body systems to small quantum devices. Simulating quantum matter and renormalization group flows is arguably one of the most "truly quantum" tasks a device can perform, as it mirrors the underlying physical geometry of the entanglement. The approach is applicable for most many-body systems of interest and applications for higher-dimensional systems are within reach.

Referee 1: For the reasons above, mostly 1) and 2), I find this work as a technically well-executed analysis of a quantum-assisted classical algorithm for the study of critical properties of the conventional Ising model, based on established methodologies and extending previously demonstrated (or proposed) analogous approaches on the same model. The work is mostly of value for those studying/testing these approaches, and thus I see it more appropriate to publish it in a specialized journal.

Response: We hope that the clarifications concerning points 1) and 2) and newly added simulations will potentially soften your concerns regarding the significance of the work. We believe that your support for publication would strongly encourage us and others to pursue the important future directions highlighted by you, including nonequilibrium dynamics and extensions to 2D systems. These directions require higher gate fidelities and larger qubit numbers, which will be enabled by sympathetic cooling of $^{171}\mathrm{Yb}^+$ qubit ions using co-trapped $^{172}\mathrm{Yb}^+$ ions in our setup (Ref. 67).

Referee 1: I have a few remarks on the content:

- What are the main reasons why MPS-based approaches are not applicable or useful here? I believe mentioning MPS ansatzes and discussing a comparison with them is necessary here, even if this has been done already in the literature. What features of MPS do not allow to implement a scheme similar to the one used in this work? The absence of causal structure? a more limited compression? the scaling of certain quantities? A paragraph explaining why a MERA-based computation for this problem is advantageous over MPS (if that is true) is necessary.

Response: MPS is another powerful variational ansatz, but it possesses a fundamentally different causal structure from MERA. In particular, an exact MPS has a strict sequential geometry, while MERA has a hierarchical structure. Consequently, when implemented as quantum circuits, evaluating local observables in an exact MPS intrinsically requires a circuit depth that scales linearly with the system size L. By contrast, the circuit depth for the same evaluation in MERA scales only logarithmically, as $O(\log L)$.

Moreover, accurately representing critical ground states and their associated logarithmic scaling of entanglement entropy requires the bond dimension of an MPS to grow polynomially with system size L. In contrast, MERA can efficiently encode such critical states with a constant bond dimension, owing to its hierarchical structure, as demonstrated in this work. This distinction is particularly important for experimental implementations.

Lastly, MPS approaches are limited to 1D systems in the sense that required bond dimensions increase exponentially in the system size for higher dimensions. In contrast, MERA capture area-law states in all dimensions with size-independent bond dimensions.

We have added a paragraph in the Discussion section to explicitly discuss why a MERA-based computation is advantageous over an MPS-based approach for the problem studied here, and to clarify the role of causal structure and entanglement scaling in enabling this advantage.

Referee 1: - In Sec. IIIA, the authors show properties in the symmetry broken phase with nonzero magnetization $\langle X \rangle$. However, in a symmetry broken phase there are two degenerate ground states. It is not clear how one of the two ferromagnetic ground states is attained instead of a generic superposition. A comment should be given.

Response: The variational optimization of the MERA with random initialization does not converge to a superposition of the two symmetry-broken ground states but instead spontaneously selects one of them: The fixed bond dimension limits the amount of entanglement that the MERA ansatz can encode. As the formation of a superposition of two symmetry-broken states increases the entanglement, the optimization

will remove one of the components to achieve a better energy accuracy for the given bond dimension. Which of the two symmetry-broken states is obtained depends on the initialization.

We have added a comment in the manuscript to clarify this point, and also updated Fig. 1 by replacing $\langle X \rangle$ with m^x to represent its absolute value.

Referee 1: - In Fig. 2b, the inset shows data indicating a critical exponent 1/8. In particular, the authors state in the text "The fitted values gradually converge to the theoretical prediction of 1/8", but I cannot see this well represented by the data shown, I actually do not see any convergence or actual quantitative correspondence with the critical value expected. I find it hard to conclude anything from the data shown.

Response: We thank the referee for pointing this out and have revised the text to provide a more careful description.

The data in Fig. 2b shows a clear power law decay of the order parameter which levels off very close to the critical point $g = 1$ due to the limited bond dimension ($\chi = 2$). The inset showed information about the fitting procedure (fits over different g regions), which is not really necessary and even confusing, as shown by the referee's reaction (one would not expect "convergence" in g_{cut}). In the updated manuscript, we simplified the presentation, removing the inset and adding instead an explicit single fit line for the experimental data. For this fit, we simply exclude the last two data points (those closest to $g = 1$), yielding an extracted critical exponent of $\beta = 0.117(4)$, which is close to the theoretical prediction of $1/8 = 0.125$ for the Ising universality class. The excluded last two points are determined by directly observing which points significantly deviate from the power-law scaling trend of the experimental data. We note that a critical exponent of $\beta = 0.123(6)$, in agreement with the prediction for the Ising criticality class, is obtained by further excluding the preceding two data points.

Response to Report of Referee 2

Referee 2: To my knowledge, this is the first time that this approach has been implemented experimentally. The results look promising to me. They should be of interest to a wide range of researchers working in this field and can in principle be implemented on any experimental platform for digital quantum simulation.

Response: We thank the referee for these positive and encouraging comments.

Referee 2: For the model system investigated, exact results are known, which enables a comparison of the experimental findings with the ground truth. While computer simulations based on MERA circuits/states can be efficiently implemented for 1d systems, the authors stress that MERA representations of two-dimensional models come with high numerical costs of tensor contractions. The hope motivating the present experiments is that improved versions of the latter might have an edge over computer simulations of 2d models by swapping tensor contractions against quantum measurements. Whether this approach will work depends on the difficulty of variationally optimizing the quantum circuits that encode the MERA states. This is a question that the current manuscript does not answer. For this reason, I would have liked to learn about how difficult it would have been to find the MERA states representing ground-states of the 1d Ising model experimentally. While I would find it unreasonable to ask the authors to have their experiment answer this question, I feel that it would really strengthen this manuscript if the variational optimization of such a circuit was simulated numerically. How many quantum states would one have to generate in order to find the optimized gate sequence used in the experiments? Can one estimate how this number would scale when moving to a 2d system with a size where one could hope of experiments to predict system properties more precisely than numerical simulations?

Response: We thank the referee for this insightful high-level summary and for raising this important question. In response, we have included numerical simulations for the variational optimization of MERA circuits in the revised manuscript (Supplementary Note 2), and we reproduce the plot here as Fig. R2.

As a concrete example, we consider the critical point $g = 1.0$ of the one-dimensional Ising model and apply a scale-invariant MERA ansatz. The true ground state at criticality is strongly correlated and challenging to approximate. Nevertheless, a scale-invariant MERA with the smallest bond dimension $\chi = 2$ can already represent this state rather accurately, achieving an energy-density error as low as 1.4×10^{-3} in noiseless numerical simulations.

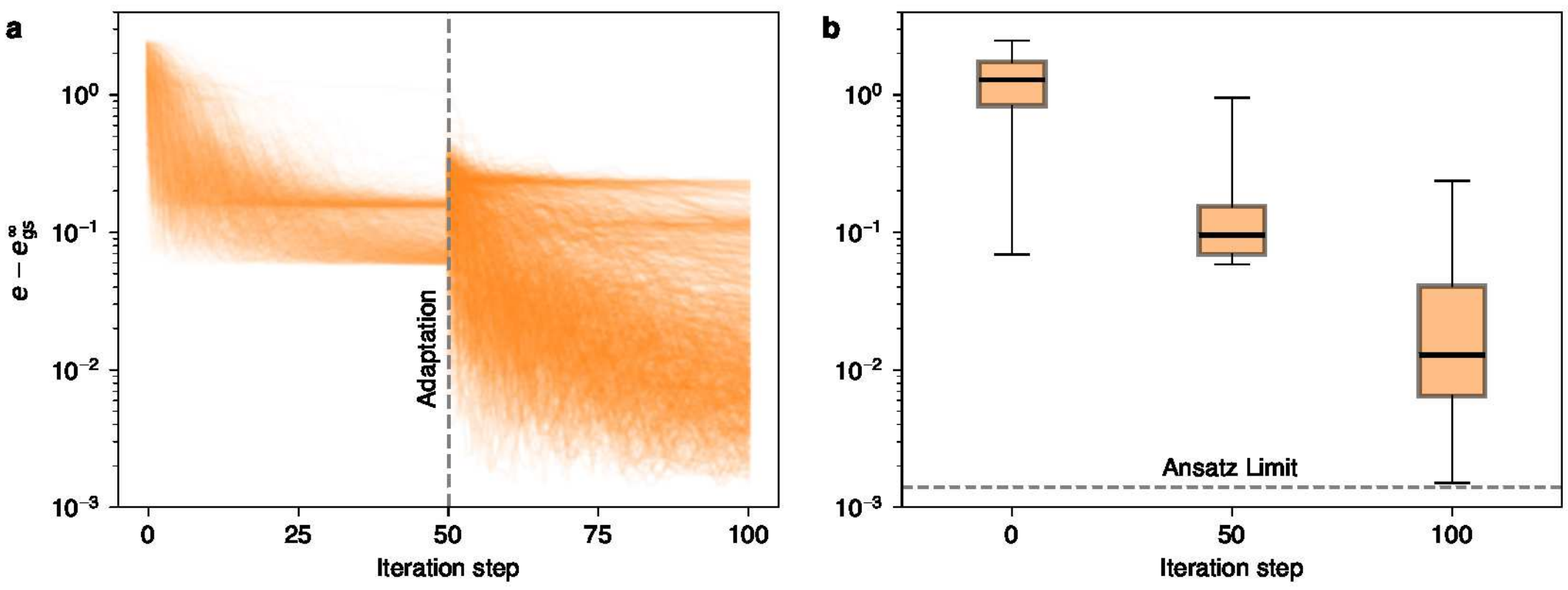

Figure R2: **Simulation of variational optimization.** (a) Optimization trajectories of the energy-density error $e - e_{\rm gs}^{\infty}$ versus iteration step for 1,000 randomly initialized MERA circuits. (b) Quantile distributions at different optimization steps. The whisker end caps indicate the minimum and maximum values, the box edges denote the 25% and 75% quantiles, and the black line marks the median. The gray dashed line indicates the ansatz limit after adaptation (second stage). [Reproduced from Fig. S2 in the updated manuscript (Supplementary Note 2).]

To reflect experimental conditions, the variational optimization simulations explicitly incorporate measurement uncertainty by sampling measurement outcomes rather than using exact energies. To reduce the sampling complexity, we employ the SPSA (Simultaneous Perturbation Stochastic Approximation) optimizer instead of standard gradient descent, which requires evaluating the energy at only two parameter points per iteration.

Figure R2a shows optimization trajectories of the energy-density error versus iteration step for 1,000 randomly initialized MERA circuits. To improve convergence and reduce resource costs, we adopt an adaptive strategy. During the first 50 iterations, we optimize a single-layer MERA ($T = 1$) using only 100 shots per measurement basis. Since the Ising Hamiltonian only involves the Z and X bases, each iteration requires 400 shots in total to evaluate the gradient and determine the move direction. In this stage, the energy error typically reaches the 0.1–0.2 range, consistent with the shot-noise limit. In the subsequent 50 iterations, we increase the number of MERA layers to $T = 6$ and the shot count to 10,000 per measurement basis. By the end of this stage, most trajectories converge to energy errors on the order of 10^{-2}, again consistent with shot-noise limit. Fig. R2b shows the corresponding quantile distributions at different optimization steps. At the end of optimization, at least 25% of the trajectories achieve an energy error below 0.01 and, in the best cases, the optimization reaches the intrinsic ansatz limit.

While it remains challenging to make quantitative predictions for the optimization cost of MERA circuits in two-dimensional systems, these one-dimensional results suggest that, with appropriate optimizer choices and warm-up strategies, the number of quantum states required for optimization is comparable to the shot count needed to reach a desired target accuracy.

Referee 2: Apart from this, I have hardly any comments:

I would have like to find a bit more information about the extraction of critical exponents in Fig. 2b. Was a fit of the log of the mean magnetization vs the log of $1 - g^2$ used?

Response: The critical exponent is extracted by fitting the measured mean magnetization to the form $A\,(1 - g^2)^{\beta}$, where β is the critical exponent and A is a constant prefactor. We have added corresponding details in the revised manuscript.

Referee 2: Fig. 1d shows two different ion-to-qubit mappings but does not explain why they are expected to be optimal for the measurement of a particular quantity. Also, I couldn't figure out why in 1a and d some of the qubit numbers are printed in gray and others in black.

Response: We determine the ion-to-qubit mapping for each circuit to reduce the estimated error for the target observable, based on the measured infidelities of the two-qubit gates:

We first aim to suppress crosstalk by avoiding the assignment of contiguous nearest-neighbor ions as measured qubits. In both Maps I and II, measured ions (qubits indicated by black circles with colored face) are therefore never nearest neighbors. Second, we aim to place higher-fidelity gates closer to the measurement side of the MERA causal-cone circuit, as gates acting near the measurement directly affect the measurement outcomes. For measurements of local observables, higher-fidelity gates are assigned to the later stages of the quantum circuit (i.e., the lower layers of the MERA circuit), since these gates act closest to the final measurement. By contrast, for measurements of entanglement entropy, high-fidelity gates are preferentially placed along the measured boundary/edge of the MERA circuit.

Based on these considerations and experimental constraints, we choose Map I in Fig. 1d for the measurement of local observables and Map II for the investigation of entanglement entropy.

In Figs. 1a and 1d, qubits shown in black are actively used in the 3-layer MERA causal-cone circuit, whereas qubits shown in gray are not used in this demonstration example. In the revised version, qubits 0-7 indicated by black circles in Fig. 1a correspond to Map I in Fig. 1d. In Map II, qubit 3 is not used (shown in gray), since it lies outside the causal cone of the measured qubits 5, 6, and 7 and is therefore not required for extracting the target entanglement entropy.

Note that, in the experiment, we used up to 12 qubits to prepare MERA causal-cone circuits with up to 5 layers; Fig. 1 presents only a 3-layer example for illustrative purposes.

Referee 2: Overall, the results are convincing and significant. With the above clarifications and a brief trainability analysis, I would strongly support publication.

Response: We thank the referee for these encouraging and positive comments. With the above clarifications and the newly added trainability analysis, we believe that the revised manuscript is significantly strengthened.

Response to Report of Referee 3

Referee 3: This manuscript demonstrates the first observation of log-law scaling entanglement entropy at the quantum critical point of the Ising model, achieved through the implementation of MERA and holographic tomography. The results are supported by a thoughtful error model that closely aligns noisy simulations with experimental data, while the measured critical scaling and entanglement transition consistently reflect Ising universality predictions. The work is significant for overcoming fundamental finite-size limitations in quantum simulation and establishing a scalable framework for studying strongly-correlated systems. The analytical approach effectively exploits MERA's causal cone structure and scale-invariance properties to enable efficient characterization of critical phenomena with logarithmic resources, presenting a coherent and powerful framework for quantum many-body simulation. Methodologically, it leverages a fully-connected trapped-ion architecture with optimized qubit mappings and advanced gate control.

Response: We thank the referee for these thoughtful and positive comments.

Referee 3: However, additional details on experimental calibration and optimization procedures would further strengthen validity and clarity. I believe this manuscript is of broad interest and qualified to be published in nature communications. To increase the readability, I hope the authors could address the points below.

Response: We thank the referee for these encouraging comments. In response, we have provided additional details on the experimental calibration and optimization procedures to strengthen the validity and clarity of the manuscript. Please see our responses below.

Referee 3: 1. In Fig. 1a, do the circled numbers indicate the execution sequence of the quantum circuit used to prepare the MERA state? If so, why does this execution order not follow a layer-by-layer construction? In Fig 1.d, do the circled numbers have the same meaning as those in Fig. 1a? What does the background color of each circle signify? A more detailed explanation of these annotations would greatly assist readers unfamiliar with the MERA formalism in deciphering these complex diagrams.

Response: The circled numbers do not indicate the execution sequence of the quantum circuit; instead, they label the qubit indices. In Fig. 1a, the MERA causal-cone circuit involves eight qubits, labeled from 0 to 7 from left to right. A more explicit quantum circuit representation is provided in Fig. S1 in Supplementary Note 1, where the qubit indices and the layer-by-layer construction are shown more concretely.

The circled qubit numbers are consistent across all subfigures in Fig. 1 as well as in Fig. S1. The background (face) color of a circled qubit indicates that the qubit is measured, and the color corresponds to the last tensor (rectangular block) acting on that qubit. For example, on Map I in Fig. 1d, qubits 3 and 4 are measured after applying the last entangling gates (blue rectangles). Qubits shown with black circles and a white background are used in the circuit but not measured, whereas qubits shown in gray circles are not used in the corresponding mapping.

We have added a more detailed explanation of these annotations in the figure captions to improve readability for readers unfamiliar with the MERA formalism.

Referee 3: 2. More detailed descriptions of the experimental implementation would improve the clarity of the research. Specifically: (1) How is the strength of the transverse field g accurately calibrated and controlled in the setup? (2) How are the parameters of the disentangler and isometry optimized, and how is the execution order of the quantum circuit determined? What key factors—such as noise resilience, resource constraints, or convergence criteria—are considered during the optimization process? (3) How are ion reset and reinitialization procedures utilized across successive experimental runs to ensure comprehensive coverage of distinct causal cones? Furthermore, what is the cumulative impact of such reset-reinitialization cycles on the overall experimental error budget?

Response: (1) In MERA circuits, the gate rotation angles are determined via classical optimization for each given value of g. Experimentally, we actively calibrated the rotation angle of single-qubit gates, the rotation angle, blue-and-red sideband imbalance and Stark shift of selected two-qubit gates before implementing the circuits.

(2) The parameters of the disentanglers and isometries are optimized using a standard variational approach, in which the gate angles are treated as variational parameters and optimized to minimize the expectation value of the target Hamiltonian. In the classical optimization procedure, convergence is reached when the energy density no longer decreases and fluctuates only within a small tolerance (typically on the order of 10^{-8} or below). We find that the optimization process is robust and yields consistent results across different initial conditions.

The execution order of the quantum circuit follows the causal-cone structure of the MERA ansatz. Gates that are logically parallel are automatically scheduled by the compiler. The MERA circuit has been shown to be intrinsically resilient to noise (Ref. 29) and requires only logarithmic circuit depth with system size. In the current setup, questions about noise resilience and resource constraints do not enter the MERA optimization, apart from choosing an experimentally feasible bond dimension.

(3) Although the MERA circuit in principle supports in-shot qubit reset, this functionality is not used in the present experimental implementation. Considering shot-by-shot reset, the ions are reset and reinitialized to the desired initial state $|0\rangle$ using optical pumping and state-preparation procedures before the next run. These reset-reinitialization cycles do not accumulate error coherently across shots, and their contribution to the overall error budget is state preparation and measurement (SPAM) errors, which are discussed in detail in the Methods section.

We have improved the Methods section and Supplementary Note accordingly with more detail.

Referee 3: 3. The authors observe that the small bond dimension χ leads to saturation of the order parameter near criticality (Fig. 2b). Besides, the finite correlation length resulting from the limited number of layers T may also contribute to the observed deviations. The manuscript would be strengthened by a more explicit discussion of both the robustness of qualitative conclusions, such as the logarithmic scaling of entropy, under these limitations, and the fundamental constraints on quantitative accuracy imposed by finite χ and T.

Response: The onset of the order-parameter near the phase transition is already astonishingly sharp and second-order phase transitions have to our knowledge not been previously demonstrated on quantum computers with such clarity.

Increasing the number of MERA layers beyond $T = 5$ does not further improve the order parameter. The saturation of the order parameter near criticality observed in Fig. 2b is dominated by the limited bond dimension $\chi = 2$. As discussed in the second-to-last paragraph of Sec. III A, further improvements can be achieved by increasing the bond dimension from $\chi = 2$ to $\chi = 4$, .

The qualitative conclusion of logarithmic scaling of entanglement entropy is robust. Concretely, the optimized scale-invariant MERA with $\chi = 2$ yields an extracted central charge of approximately $c \approx 0.475$, which is close to the theoretical value $c = 1/2$ (see the Methods section). As expected, the central charge obtained from scale-invariant MERA converges toward 1/2 as the bond dimension χ is increased.

Regarding the quantitative accuracy of the MERA simulation, we find that the error in the energy density is $\lesssim 10^{-3}$ for $\chi = 2$ and $T \leq 5$. For local observables, such as nearest-neighbor correlations and on-site field expectation values, the deviations between simulation and theory are significantly smaller than the experimental uncertainties, as shown in Fig. 1c. While increasing χ and T can reduce numerical errors by orders of magnitude, in our experiment the dominant limitation on quantitative accuracy arises from experimental shot noise rather than from the MERA truncation.

We have revised the manuscript accordingly to increase the clarity of the research.

III List of Changes

- Added numerical simulations and analysis of the 1D XXZ spin chain (Supplementary Note 4) to demonstrate the viability of our MERA approach for a more complex model with a Berezinskii-Kosterlitz-Thouless (BKT) phase transition.
- Included a detailed analysis and numerical simulations of the variational optimization process to address the trainability and resource overhead of MERA circuits (new Supplementary Note 2).
- Added a paragraph in the Discussion section to explicitly compare MERA with MPS.
- Revised Fig. 2b and the corresponding main text to clarify the extraction of the critical exponent.
- Expanded the caption of Fig. 1 to provide detailed explanations of the annotations, including the circled qubit indices, background colors. Also added an arrow in the figure to make the preparation direction obvious.
- Other clarification improvements.
